# Prevention by Dietary Polyphenols (Resveratrol, Quercetin, Apigenin) Against 7-Ketocholesterol-Induced Oxiapoptophagy in Neuronal N2a Cells: Potential Interest for the Treatment of Neurodegenerative and Age-Related Diseases

**DOI:** 10.3390/cells9112346

**Published:** 2020-10-23

**Authors:** Aline Yammine, Amira Zarrouk, Thomas Nury, Anne Vejux, Norbert Latruffe, Dominique Vervandier-Fasseur, Mohammad Samadi, John J. Mackrill, Hélène Greige-Gerges, Lizette Auezova, Gérard Lizard

**Affiliations:** 1 Team Bio-peroxIL, “Biochemistry of the Peroxisome, Inflammation and Lipid Metabolism” (EA7270), University Bourgogne Franche-Comté, Inserm, 21000 Dijon, France; alineyammine5@gmail.com (A.Y.); thomas.nury@u-bourgogne.fr (T.N.); anne.vejux@u-bourgogne.fr (A.V.); norbert.latruffe@u-bourgogne.fr (N.L.); 2 Bioactive Molecules Research Laboratory, Doctoral School of Sciences and Technologies, Faculty of Sciences, Lebanese University, Fanar, Jdeidet P.O. Box 90656, Lebanon; hgreige@ul.edu.lb (H.G.-G.); auezova_l@hotmail.com (L.A.); 3 Faculty of Medicine, LR12ES05, Lab-NAFS ‘Nutrition-Functional Food & Vascular Health’, University Monastir, 5019 Monastir, Tunisia; zarroukamira@gmail.com; 4 Faculty of Medicine, University Sousse, 4000 Sousse, Tunisia; 5 Team OCS, Institute of Molecular Chemistry of University of Burgundy (ICMUB UMR CNRS 6302), University of Bourgogne Franche-Comté, 21000 Dijon, France; dominique.vervandier-fasseur@u-bourgogne.fr; 6 LCPMC-A2, ICPM, Depterment of Chemistry, University Lorraine, Metz Technopôle, 57070 Metz, France; mohammad.samadi@univ-lorraine.fr; 7 Department of Physiology, School of Medicine, University College Cork, T12 Cork, Ireland; J.Mackrill@ucc.ie

**Keywords:** 7-ketocholesterol, oxysterol, apigenin, quercetin, resveratrol, oxiapoptophagy, polyphenol, age-related diseases, N2a cells

## Abstract

The Mediterranean diet is associated with health benefits due to bioactive compounds such as polyphenols. The biological activities of three polyphenols (quercetin (QCT), resveratrol (RSV), apigenin (API)) were evaluated in mouse neuronal N2a cells in the presence of 7-ketocholesterol (7KC), a major cholesterol oxidation product increased in patients with age-related diseases, including neurodegenerative disorders. In N2a cells, 7KC (50 µM; 48 h) induces cytotoxic effects characterized by an induction of cell death. When associated with RSV, QCT and API (3.125; 6.25 µM), 7KC-induced toxicity was reduced. The ability of QCT, RSV and API to prevent 7KC-induced oxidative stress was characterized by a decrease in reactive oxygen species (ROS) production in whole cells and at the mitochondrial level; by an attenuation of the increase in the level and activity of catalase; by attenuating the decrease in the expression, level and activity of glutathione peroxidase 1 (GPx1); by normalizing the expression, level and activity of superoxide dismutases 1 and 2 (SOD1, SOD2); and by reducing the decrease in the expression of nuclear erythroid 2-like factor 2 (Nrf2) which regulates antioxidant genes. QCT, RSV and API also prevented mitochondrial dysfunction in 7KC-treated cells by counteracting the loss of mitochondrial membrane potential (ΨΔm) and attenuating the decreased gene expression and/or protein level of AMP-activated protein kinase α (AMPKα), sirtuin 1 (SIRT1) and peroxisome proliferator-activated receptor γ coactivator-1α (PGC-1α) implicated in mitochondrial biogenesis. At the peroxisomal level, QCT, RSV and API prevented the impact of 7KC by counteracting the decrease in ATP binding cassette subfamily D member (ABCD)3 (a peroxisomal mass marker) at the protein and mRNA levels, as well as the decreased expresssion of genes associated with peroxisomal biogenesis (*Pex13*, *Pex14*) and peroxisomal β-oxidation (*Abcd1*, *Acox1*, *Mfp2*, *Thiolase A*). The 7KC-induced decrease in ABCD1 and multifunctional enzyme type 2 (MFP2), two proteins involved in peroxisomal β-oxidation, was also attenuated by RSV, QCT and API. 7KC-induced cell death, which has characteristics of apoptosis (cells with fragmented and/or condensed nuclei; cleaved caspase-3; Poly(ADP-ribose) polymerase (PARP) fragmentation) and autophagy (cells with monodansyl cadaverine positive vacuoles; activation of microtubule associated protein 1 light chain 3–I (LC3-I) to LC3-II, was also strongly attenuated by RSV, QCT and API. Thus, in N2a cells, 7KC induces a mode of cell death by oxiapoptophagy, including criteria of OXIdative stress, APOPTOsis and autoPHAGY, associated with mitochondrial and peroxisomal dysfunction, which is counteracted by RSV, QCT, and API reinforcing the interest for these polyphenols in prevention of diseases associated with increased 7KC levels.

## 1. Introduction

With increased lifespans in human populations, the number of age-related diseases (cardiovascular diseases, eye diseases (cataract), age-related macular degeneration (AMD or ARMD)), neurodegenerative diseases (Parkinson’s disease, dementias and Alzheimer’s disease (AD), certain cancers) increase [1,2,3]. As these diseases have an important societal and financial impact, it is imperative to find drugs to decrease their frequencies. It is therefore important to better know the physiopathology of these diseases and their associated molecular mechanisms, as well as the factors contributing to their initiation and development. Such knowledge will facilitate the identification of nutraceutical and pharmacological approaches for the prevention and curation of age-related diseases [4]. Among non-pharmacological approaches, the most efficient interventions include limiting caloric or protein intake and increasing aerobic exercise. Dietary patterns including the Mediterranean and Okinawan diets, which are rich in polyphenols and omega-3 fatty acids, are also associated with improved age-related health [5]. Many of the components of the Mediterranean diet, such as fruit, vegetables oil and red wine, are rich in polyphenols [6,7,8]. As a result, several studies have been carried out to clarify the role of polyphenols on aging and age-related diseases, that are characterized by enhanced oxidative stress, mitochondrial and peroxisomal dysfunction which progressively trigger lipid peroxidation, protein carbonylation, DNA damage, senescence, and cell death, favoring tissue injury to vessels, heart, brain, liver, muscles, and bones [9,10,11,12]. Polyphenols may also have the property of crossing the blood–brain barrier under certain conditions [13,14], which makes them attractive for targeting nerve cells of the central nervous system and treating neurodegenerative diseases. In addition, polyphenols such as resveratrol and apigenin are now considered as neurotrophines since, on nerve cells, they have both anti-oxidant and differentiating properties [15,16,17]. In addition, resveratrol has been shown to trigger muscle differentiation on mouse-skeletal muscle-derived C2C12 myoblasts [18]. Altogether, these data support that polyphenols have gerontoprotective activities and could be also of interest in preventing frailty syndrome: this is a clinical syndrome in older adults that carries an increased risk for poor health outcomes including falls, incident disability, hospitalization, and mortality [19]. In addition, in the human Adult Retinal Pigment Epithelial cell line-19 (ARPE-19) which is a suitable in vitro model for screening therapeutics against AMD [20], we previously reported that resveratrol (RSV) was able to prevent 7-ketocholesterol (7KC)-induced cytotoxicity [21].

Among cholesterol oxide derivatives (oxysterols) obtained by cholesterol oxidation [22], 7KC (also named 7-oxocholesterol) is mainly formed by cholesterol auto-oxidation [23,24,25]. In the body, 7KC can be supplied by food. It is further delivered to vascular cells via Low Density Lipoproteins (LDL) and is eliminated by the liver in the form of bile acids [26]. The accumulation of 7KC in the tissues mainly results from stress conditions (overproduction of reactive oxygen and nitrogen species (ROS, RNS), presence of metals (Fe^2+^, Cu^2+^, etc.) leading to the Fenton and Haber–Weiss reactions) [27]. Although the biogenesis pathway of 7KC by auto-oxidation is considered to be preponderant [28], 7KC can also be formed enzymatically by the enzyme 11β-HSD2 from 7β-hydroxycholesterol in some cells and from 7-dehydrocholesterol by the enzyme CYP7A1 [29]. Therefore, to oppose the detrimental effects of 7KC (oxidative stress, inflammation and cell death), it is judicious to find molecules opposing these cytotoxic effects, or strategies that contribute to its biodegradation [29,30], rather than by using enzyme inhibitors.

At the moment, several studies suggest that 7KC plays a key role in the most common age-related diseases: cardiovascular diseases, eye diseases (cataract, AMD) and Alzheimer’s disease (AD) [31,32,33,34]. 7KC is present at elevated levels in oxidized LDL and in the atherosclerotic plaques of patients with cardiovascular diseases [35], in drusens present in the retinas of patients with AMD [36], and in the cortex of AD patients [34]. Unfavorable environmental factors such as air pollution can interact with the deleterious effects of 7KC [37,38], and when associated with iron nanoparticles, the toxic effects of 7KC are exacerbated [39]. At the cellular level, it is well established that 7KC accumulates in lipid rafts and induces various cytotoxic effects at high concentrations (25–50 µM): oxidative stress and organelle dysfunction (endoplasmic reticulum, lysosome, mitochondria and peroxisome), which contribute to activate apoptosis and autophagy [25]. Based on these different characteristics, 7KC-induced cell death has been defined as oxiapoptophagy (OXIdative stress + APOPTOsis + autoPHAGY) [40,41]. In human aortic smooth muscle cells, 7KC-induced autophagy is a cellular protective response that attenuates 7KC-induced cell death [42]. At the moment, 7KC-induced oxiapoptophagy has been described in human promonocytic U937 cells [43], 158N murine oligodendrocytes [44], BV-2 microglial cells [45] and in bone marrow mesenchymal stem cells [46]. In addition, 7KC is an inducer of inflammation involved in the progression of several age-related diseases; it favors the expression of adhesion molecules (E-selectin, intracellular cell adhesion molecule-1 (ICAM-1) and vascular cell adhesion molecule 1 (VCAM-1) proteins) [47], pro-inflammatoty cytokines (interleukine (IL)-1β, IL-6 and IL-8) [48,49,50] and enhances the mRNA expression of lipoxigenases and cyclooxigenases [51]. In ARPE-19 cells, 7KC-induced inflammation is mostly mediated though the Toll Like receptor 4 (TLR4) with some cross-activation of Epidermal Growth Factor Receptor (EGFR)-related pathways [52].

Since 7KC induces oxidative stress, inflammation and cell death which contribute to the physiopathology of age-related diseases, it is important to identify molecules capable of counteracting these cytotoxic effects. Only a few natural and synthetic molecules, as well as mixtures of molecules (oils, plant extracts), have been shown efficiently prevent 7KC-induced toxicity [30]. These natural molecules include anti-oxidants (reduced glutathione (GSH), indicaxanthine (a bioactive pigment from cactus pear fruit: α-tocopherol)) and fatty acids (docosahexaenoic acid (DHA, C22:6 n-3); oleic acid (C18:1 n-9)). Among these, α-tocopherol is the most potent cytoprotective agent in several types of cells [30]. Currently, *N*-acetyl-cysteine (NAC) as well as dimethylfumarate, and its major metabolite (monomethylfumarate), two inducers of the nuclear erythroid 2-like factor 2 (Nrf2) metabolic pathway [53,54], are the only synthetic molecules demonstrated to prevent 7KC- or 7β-hydroxycholesterol-induced oxiapoptophagy [55,56]. Mixtures of lipids (olive, argan and milk thistle seed oils) as well as Xuezhikang (red yeast rice extract) also have strong cytoprotective activities [30,57]. However, few data are available on the ability of polyphenols to prevent 7KC-induced cytotoxicity [58]. When an oxysterol mixture including 7α-hydroxycholesterol, 7β-hydroxycholesterol, 7-KC, cholesterol 5α,6α-epoxide, cholesterol 5β,6β-epoxide, cholestane-3β,5α,6β-triol, and 25-hydroxycholesterol was used on intestinal cells or human peripheral blood mononuclear cells, protective effects of Cocoa bean shells (containing a high level of epicatechin) [59], and of olive oil polyphenols [60,61] were revealed. In addition, when differentiated murine PC12 cells and human neuroblastoma SH-SY5Y cells were treated with taxifolin (dihydroquercetin), it was shown that 7KC-induced neuronal apoptosis was prevented by suppressing the Akt and NF-κB activation mediated cell death [62]. At the moment nothing is known on the ability of polyphenols to prevent 7KC-induced oxiapoptophagy, which is a particular type of cell death induced by cytotoxic oxysterols [40]. So, we chose to evaluate the cytoprotective activities of three polyphenols widely represented in the Mediterrannean diet (resveratrol (RSV), quercetin (QCT) and apigenin (API)) [63]. Polyphenols include two classes: flavonoids and non-flavonoids. RSV (corresponding to trans-RSV which is biologically active) is a non-flavonoid polyphenol of the stilbenic class; it is present in grapes, blackberries or peanuts and it is found in significant quantities in red wine. QCT is a flavonoid of the flavonol type found in many fruits and vegetables. API belongs to the flavone family, a subclass of flavonoids; it is found in high amounts in parsley, rosemary, celery and chamomile. QCT has been shown to activate both Liver X Receptor (LXR)α and LXRβ, whereas API only activates LXRβ [64]. Since polyphenols could cross the blood–brain barrier [13,14], and act as chemoprotective agents by interacting with the pathomechanisms of several age-related diseases, including neurodegenerative diseases [30,32,33,65,66], it was important to define whether RSV, QCT and API were able to prevent 7KC-induced oxiapoptophagy.

To this end, murine neuroblastoma N2a cells, which have been widely used to study neuronal differentiation, axonal growth and signaling pathways [16,17,67] were chosen as a model to evaluate and characterize the cytotoxic effects of 7KC (ROS overproduction and oxidative stress, mitochondrial and peroxisomal dysfunction, apoptosis and autophagy) and the cytoprotective activities of RSV, QCT and API.

## 2. Materials and Methods

### 2.1. Cell Culture and Treatments

The mouse neuro-2a (N2a) neuroblastoma cell line (Ref: CCL-131, American Type Culture Collection (ATCC), Manassas, VA, USA) was maintained in Dulbecco’s modified Eagle medium (DMEM, Lonza, Amboise, France) containing 10% (v/v) of heat-inactivated fetal bovine serum (FBS) (Pan Biotech, Aidenbach, Germany) (30 min, 56 °C) and 1% (v/v) of penicillin (100 U/mL)/streptomycin (100 mg/mL) (Pan Biotech). The cells were incubated at 37 °C in a humidified atmosphere (5% CO_2_, 95% air) and passaged twice a week. The cells were seeded for the different experimental conditions, at a density of 1.2 × 10^5^ cells per well containing 1 mL of culture medium with 10% FBS in 6-well plates (FALCON, Becton Dickinson, Franklin Lakes, NJ, USA) or in Petri dishes at 30,000 cells/cm^2^ (100 mm diameter) in order to assess the ability of trans-resveratrol (RSV), quercetin (QCT) and apigenin (API) to counteract the cytotoxicity induced by 7-ketocholesterol (7KC). 7KC (ref: C2394; purity >90%), RSV (trans-resveratrol, ref: 501-36-0; purity 99%) and QCT (ref: #Q0125; purity >98%) were from (Sigma-Aldrich, St Quentin-Fallavier, France); API was from (Euromedex, Souffelweyersheim, France; ref: #52262; purity >97%). The stock solution of 7KC was prepared at 800 µg/mL (2 mM) as previously described [68]. The stock solutions of polyphenols were prepared as follows: RSV at 50 mM in absolute ethanol (EtOH; Carlo Erba Reagents, Val de Reuil, France), whereas dimethyl sulfoxide (DMSO; Sigma-Aldrich) was used as vehicle to dissolve QCT and API prepared at 50 mM. Alpha-tocopherol (Sigma-Aldrich, ref: T3251; purity >96%) was used as a positive control for cytoprotection since its cytoprotective effects have been shown on several cell lines [30,33]. By using α-tocopherol as the reference cytoprotective molecule, it not only makes it possible to evaluate the cytoprotection of polyphenols (RSV, QCT, API) in relation to α-tocopherol, but also to estimate their cytoprotective activities in relation to molecules considered cytoprotective and previously compared with α-tocopherol. After 24 h of cell culture, the culture medium was removed; polyphenols (RSV, QCT and API) used at various concentrations ranging from 1.5 to 25 µM, or α-tocopherol (400 μM), were introduced in the culture medium without or with 7KC (50 µM), and the cells were incubated for 48 h. The choice of the concentration of 7KC was based on the viability tests performed in this study which show that 50 µM is the 50% inhibiting concentration (IC50) in N2a cells. Alpha-tocopherol was used at the highest non-cytotoxic concentration (400 µM) able to prevent 7KC-induced apoptosis [69]. In addition, to clarify the ability of RSV, QCT and API to prevent 7KC-induced cell death, N2a cells were also pre- and post-treated with polyphenols (1.5 to 25 µM) added 2 h before or 2 h after 7KC, respectively.

The cytoprotective activities of RSV, QCT and API against 7KC-induced cell death were also evaluated in co-treatment on human neuroblastoma cell lines SK-N-BE (American Type Culture Collection (ATCC); ATCC CRL-2271) and SH-SY5Y (ATCC CRL-2266) that were maintained in DMEM medium (Lonza) supplemented with 10% FBS (Pan Biotech) (heat-inactivated for 30 min, 56°C) and 1% (v/v) of penicillin (100 U/mL) / streptomycin (100 mg/mL) (Pan Biotech). The SK-N-BE and SH-SY5Y cells were seeded at a density of 200,000 cells and 250,000, respectively, per well containing 2 mL of culture medium with 10% FBS in 6-well plates in the presence or absence of polyphenols (1.5 to 25 µM), with or without 7KC (50 µM).

To study the mechanism of autophagy (lethal or survival autophagy) associated with 7KC in N2a cells, rapamycin (Sigma-Aldrich; ref: #37094) and 3-methyladenine (3-MA) (Sigma-Aldrich; ref: #M9281) were prepared at 1 mM and 100 mM in DMSO, respectively, and stored at −20 °C. Rapamycin (500 nM) was used as an inducer of autophagy: it was simultaneously added to the culture medium with or without 7KC (50 µM). 3-MA, widely used as an autophagy inhibitor, was also simultaneously added to the culture medium with or without 7KC (50 µM).

### 2.2. Measurement of Cell Viability with the Fluorescein Diacetate (FDA) Assay

The viability of N2a, SK-N-BE and SH-SY5Y cells was measured with fluorescein diacetate (FDA) (Sigma-Aldrich) as previously described [16,17,70]. The FDA assay evaluates the ability of living cells to convert the non-fluorescent FDA into the green fluorescent metabolite fluorescein after cleavage by cellular esterases. The measured signal serves as indicator for viable cells since FDA is a cell-permeant esterase substrate probe. It is considered that FDA positive cells correspond to viable cells, whereas FDA negative cells can be either cells with altered plasma membranes, lower esterase activities or dead cells. At the end of the treatment, cells were incubated in the dark with 15 µg/mL FDA for 5 min at 37 °C, rinsed twice with phosphate buffer saline (PBS), and then lysed with 10 mM Tris-HCl solution containing 1% sodium dodecyl sulfate (SDS) for 10 min. Fluorescence intensity was measured with excitation at 485 nm and emission at 528 nm using a TECAN fluorescence microplate reader (Sunrise spectrophotometer, TECAN, Lyon, France). All assays were performed in four independent experiments and realized in triplicate. The data were expressed as percentage of the control.

### 2.3. Evaluation of Adherent Cells by Sulforhodamine 101 Staining

As previously reported [70], sulforhodamine 101 (SR101) assay was used to assess the cytotoxic effect of 7KC and polyphenols on N2a cells at different concentrations ranging from 0.5 to 100 µM. SR101 is an anionic dye that electrostatically binds to cellular proteins [16]. SR101 permits the quantification of adherent cells, considered as viable cells, since cell death is associated with a loss of cell adhesion. The experiments were realized four times in triplicate. The data were expressed as percentage of the control.

### 2.4. Measurement of Polyphenols Uptake by Spectrofluorescence

To examine the uptake of RSV, QCT and API, fluorescence measurements were performed on N2a cells incubated with the polyphenols at various concentrations (3.125, 6.25, 12.5 and 25 µM) and times (3, 24 and 48 h), based on the fluorescence properties of polyphenols [71,72,73]. First, the λEx_max_ of RSV, QCT and API were determined by spectrofluorescence with a TECAN fluorescence microplate reader (Sunrise spectrophotometer, TECAN). After fixing the fluorescence emission wavelength at 530 nm, the fluorescence intensity was measured in a 96-well black polystyrene microplate containing the different solutions of polyphenols (10 µM in phosphate buffer saline (PBS)). Then, to evaluate the uptake of polyphenols on N2a cells, 1 × 104 cells were cultured for 24 h at 37 °C into 96-well plates in triplicate and the polyphenols were added to the culture medium for an additional period of time (3, 24 and 48 h) at a final concentration of 3.125, 6.25, 12.5 and 25 µM. At the end of treatment, the culture medium was removed, cells were washed with PBS, and lysed with a 10 mM Tris-HCl solution containing 1% sodium dodecyl sulfate (SDS) for 10 min. For each polyphenol, the fluorescence intensity was measured at 530 nm using the corresponding λEx_max_: RSV: 370 nm; QCT: 450 nm; API: 430 nm. Data are expressed as fold increase in the fluorescence intensity per well against the control.

### 2.5. Flow Cytometric Evaluation of Plasma Membrane Permeability and Cell Death by Staining with Propidium Iodide

Cell mortality was measured with propidium iodide (PI). PI, a hydrophilic probe, is an intercalator of nucleic acids. When excited by a blue light (488 nm), PI produces a red/orange fluorescence. PI enters in the cells with damaged, permeabilized plasma membranes which are considered as dead cells [68]. Flow cytometric evaluation of cellular permeability and cell death with PI was carried out as previously described [70]. Ten thousand cells were acquired for each sample, and the data were analyzed with FlowJo (Tree Star Inc., Ashland, OR, USA) software. All assays were performed with at least in three independent experiments.

### 2.6. Flow Cytometric Measurement of Reactive Oxygen Species(ROS) with Dihydroethidine

Dihydroethidine (DHE) is a fluorescent probe that measures ROS production (including superoxide anions (O_2_^•−^)) by flow cytometry [69,74]. Indeed, the non-fluorescent probe (DHE) is rapidly oxidized by ROS, once it enters the cell, and forms ethidium (HE) which binds to DNA and exhibits an orange/red fluorescence in response to a blue excitation (λEx Max = 488 nm; λEm Max = 575 nm). DHE (Invitrogen/Thermo Fisher Scientific, Courtaboeuf, France) was prepared at 10 mM in dimethyl sulfoxide (DMSO) and used at a final concentration of 2 µM. After 48 h of treatment with or without 7KC (50 μM) in the presence or absence of RSV, QCT or API at 3.125 or 6.25 µM, the living and dead cells were collected in a tube and then centrifuged for 5 min at 300× *g*. The pellet was resuspended in 1 mL of PBS containing 2 μM of DHE and incubated for 15 min at 37 °C. The stained cells were then analyzed using a BD Accuri™ C6 flow cytometer (Becton Dickinson, Franklin Lakes, NJ, USA); the fluorescent signals were collected through a 580 nm band pass filter. 10,000 cells were acquired from each sample. Data were analyzed with FlowJo (Tree Star Inc.) software. All assays were performed with at least three independent experiments.

### 2.7. Flow Cytometric Quantification of Cells with Depolarized Mitochondria with DiOC_6_(3)

The cationic lipophilic dye 3, 3′-dihexyloxacarbocyanine iodide DiOC_6_(3) is a probe that accumulates in the mitochondria proportionally to the value of the mitochondrial membrane potential (ΔΨm) [68]. The higher the ΔΨm, the more the probe accumulates. Thus, mitochondria with a normal ΔΨm will be more fluorescent than depolarized mitochondria, with a low ΔΨm, indicated by a decrease in green fluorescence collected through a 520 ± 10 nm band pass filter on a BD Accuri™ C6 flow cytometer. At the end of treatments, N2a cells were stained for 15 min at 37 °C with DiOC_6_(3) (Invitrogen/Thermo Fisher Scientific) used at 40 nM. For each sample, 10,000 cells were acquired, and data were analyzed with FlowJo (Tree Star Inc.) software. All assays were performed with at least three independent experiments.

### 2.8. Flow Cytometric Measurement of Mitochondrial ROS Production with MitoSOX-Red

The MitoSOX-Red mitochondrial superoxide indicator is a selective dye used for the detection of superoxide anion (O_2_^•−^) in the mitochondria [75]. In the mitochondria, this probe is oxidized by O_2_^•−^, and exhibits orange/red fluorescence (λEx = 510 nm; λEm = 580 nm). The oxidation product becomes highly fluorescent upon binding to nucleic acids. Mitochondrial production of O_2_^•−^ was quantified by flow cytometry after staining with MitoSOX-Red (Thermo Fisher Scientific) initially prepared at 5 mM in PBS. Briefly, adherent and non-adherent cells were incubated with 5 µM MitoSOX in PBS for 15 min at 37 °C. Flow cytometric analyses were immediately performed. The fluorescent signals were detected through a 580 ± 20 nm band pass filter using a BD Accuri™ C6 flow cytometer. For each sample, 10,000 cells were acquired. Data were analyzed with FlowJo software (Tree Star Inc). All assays were performed with at least three independent experiments.

### 2.9. Flow Cytometric Quantification of the Expression of ABCD3

Indirect immunofluorescence with an antibody raised against the ATP binding cassette subfamily D member (ABCD)3 peroxisomal transporter was used to detect the peroxisomes [76,77]. Adherent and non-adherent cells were collected and fixed with freshly prepared 2% (*w/v*) paraformaldehyde (Sigma-Aldrich) diluted in PBS for 15 min at room temperature. After washing in PBS, cells were incubated in PFS buffer (PBS/5% FBS/0.05% saponin (Sigma-Aldrich), 30 min, room temperature (RT)). Furthermore, the cells were washed with PBS and incubated for 1 h at RT with a rabbit polyclonal antibody raised against ABCD3 (ref: #11523651, Pierce/Thermo Fisher Scientific, Brumath, France) diluted (1/500) in PFS buffer. Cells were then washed with PBS and incubated in the dark with a goat anti-rabbit secondary antibody coupled with Alexa 488 (Santa-Cruz Biotechnology, Santa Cruz, CA, USA) diluted at 1/500 in PFS buffer (30 min in the dark, RT). After washing in PBS, the cells were resuspended in PBS and immediately analyzed by flow cytometry on a BD Accuri™ C6 flow cytometer. Fluorescence was collected from 10,000 cells through a 520 ± 20 nm bandpass filter. Absolute and conjugated controls (cells without antibodies, and without primary antibody, respectively) were performed. Data were analyzed with FlowJo software (Tree Star Inc.).

### 2.10. Quantification of Catalase Activity

Catalase, a specific peroxisomal enzyme, degrades hydrogen peroxide into water and molecular oxygen (2 H_2_O_2_ → 2 H_2_O + O_2_). Catalase (CAT) activity was determined by measuring the consumption of H_2_O_2_ at 240 nm on N2a cells cultured for 48 h with or without 7KC (50 µM) in the absence or in the presence RSV, QCT or API (3.125 µM) [45]. At the end of treatment, cells were trypsinized, washed with PBS, and lysed in radioimmunoprecipitation assay (RIPA) buffer (10 mM Tris−HCl, pH 7.2, 150 mM NaCl, 0.5% Nonidet NP40, 0.5% Na deoxycholate, 0.1% SDS, 2 mM EDTA and 50 mM NaF) in the presence of 1/25 complete protease inhibitor cocktail tablets (Roche Diagnostics, Indianapolis, IN, USA) for 30 min on ice. Cells lysates were collected after centrifugation (12,000× *g*, 20 min, and 4 °C). The reaction was initiated by the addition of Tris-HCl (1 M, pH 7.4), MilliQ H_2_O and H_2_O_2_ (400 mM) (1:1:17) and the amount of H_2_O_2_ remaining after 2 min was measured spectrophotometrically using a microplate reader (Tecan Infinite M 200 Pro) (240 nm). Catalase activity was determined based on the amount of H_2_O_2_ remaining, from a standard curve activity, and was expressed relative to the protein content. Protein was determined with the Pierce TM BCA protein assay kit (Ref: 23227; ThermoScientific, Rockford, IL, USA).

### 2.11. Quantification of Superoxide Dismutase (SOD) Activity

The activity of cellular superoxide dismutase (SOD) was measured according to the method of Beauchamp and Fridovich [78]. This method is based on the ability of the superoxide anion (O_2_^•−^) to reduce the Nitro blue Tetrazolium (NBT). Under aerobic conditions, the riboflavin, *L*-methionine and NBT mixture gives a bluish coloration. However, in the presence of SOD, the oxidation of NBT is inhibited, hence attenuating the blue coloration. The cell lysates were placed in a phosphate buffer (50 mM, pH 7.8), Ethylenediaminetetraacetic (EDTA) (0.1 mM), *L*-methionine (13 mM), riboflavin (2 μM) and NBT (75 mM). The mixture was then exposed to white light for 20 min. SOD activity was measured at 560 nm. The unit of activity is expressed as the amount of enzyme necessary to inhibit the reduction in NBT by 50%.

### 2.12. Quantification of Glutathione Peroxidase (GPx) Activity

The activity of glutathione peroxidase (GPx) was determined according to the method of Flohé and Günzler [79]. The cell lysates were incubated with 0.1 mM reduced glutathione (GSH) in phosphate buffered saline solution (50 mM, pH 7.8). The reaction was initiated by adding 50 μL of H_2_O_2_ and stopped by incubating the mixture with 250 μL of trichloroacetic acid (1% TCA) for 30 min at 4 °C, then centrifuged at 1000× *g* for 10 min. The absorbance was read by spectrophotometry at 412 nm on a Tecan Infinite M 200 Pro (Tecan, Männedorf, Switzerland). The activity of GPx is expressed in μmol of GSH/min/mg of protein. Protein content was determined with the Pierce TM BCA protein assay kit (Ref: 23227; ThermoScientific).

### 2.13. Morphological Characterization of Apoptotic Cells: Evaluation of Nuclear Morphology with Hoechst 33342

Nuclear morphology of 7KC-treated N2a cells cultured without or with polyphenols was characterized by fluorescence microscopy after staining with Hoechst 33342 (2 µg/mL) [80]. Normal cells have regular and round nuclei whereas, apoptotic cells are characterized by condensed and/or fragmented nuclei. At the end of the treatment, cells were deposited onto glass slides by cytocentrifugation (5 min, 1500 rpm) with a cytospin 2 (Shandon, Sheschire, WA7 1PR, UK), then mounted in fluorescent mounting medium (DakoCytomation, Dako, Coppenhagen, Denmark) and stored in the dark at 4 °C until observation. The slides were examined under an Axioskop right microscope (Zeiss, Jena, Germany) with ultraviolet light. A total of 300 cells per sample were counted to calculate the percentage of apoptotic cells.

### 2.14. Cytological Characterization of Autophagic Cells after Staining with Monodansylcadaverine

The presence of cytoplasmic structures corresponding to autophagic vacuoles was examined by staining with monodansylcadaverine (MDC) [81]. MDC (λEx max 340 nm, λEm max 530 nm) (Sigma) was prepared at 0.1 M in DMSO and added to the culture medium at a final concentration of 0.1 mM. After 15 min of incubation at 37 °C, N2a cells seeded on glass slides in 6-well plates, were washed and stained cells were immediately examined under an Axioskop A1 light microscope (Zeiss) by using UV light excitation. Three hundred cells were examined for each sample.

### 2.15. Protein Analysis: Polyacrylamide Gel Electrophoresis and Western Blotting

Protein analysis was realized as previously described by polyacrylamide gel electrophoresis and Western blotting [68]. After 48 h of treatment, adherent and nonadherent cells were collected, washed in PBS and lysed for 30 min on ice in a RIPA buffer (10 mM Tris-HCl, pH 7.2, 150 mM NaCl, 0.5% Nonidet NP40, 0.5% Na deoxycholate, 0.1% SDS, 2 mM EDTA and 50 mM NaF) containing a complete protease inhibitor cocktail (Roche Diagnostics, Indianapolis, IN, USA) diluted 1/25. Cell lysates were cleared by centrifugation at (20 min, 20,000× *g*) to eliminate cell debris, and the supernatant was collected. The protein concentrations were measured using bicinchoninic acid reagent (Sigma Aldrich). Seventy micrograms of protein were diluted in loading buffer (125 mM Tris-HCl, pH 6.8, 10% beta-mercaptoethanol, 4.6% SDS, 20% glycerol, and 0.003% bromophenol blue), separated on a 14% or 8% SDS-PAGE gel, then transferred onto a nitrocellulose membrane (Bio-Rad, Marne La Coquette, France). Nonspecific binding sites were blocked by incubation for 1 h with 5% milk powder in PBST (PBS, 0.1% Tween 20, pH 7.2), the membrane was incubated overnight (4 °C) with the primary antibody diluted in 5% milk PBST. For apoptosis and autophagy analysis, various antibodies were used: an antibody directed against caspase-3 for detecting endogenous levels of full length casapase-3 (35 kDa) and cleaved caspase-3 (17 kDa) (rabbit polyclonal antibody; Cell signaling/Ozymes; ref: #9662); an antibody raised against PARP for detecting endogenous level of full length PARP (110 kDa) and the 89 kDa cleaved fragment of PARP (Cell signaling), and an antibody against LC3 for detecting LC3-I (18 kDa) and LC3-II (16 kDa) (rabbit polyclonal antibody; Sigma-Aldrich, ref: L8918); these antibodies were used at 1/1000 final dilution. For the antioxidant enzyme analysis, the following antibodies were used: Nrf2 (Assay bioTech, ref: #C0279), GPx1 (GeneTex, ref: #GTX116040-S), SOD1 (GeneTex, ref: #GTX100554-S), and SOD2 (GeneTex, ref: #GTX116093-S). These rabbit polyclonal antibodies were used at a final dilution of 1/1000. In addition, the polyclonal goat antibody detecting catalase (R&D systems, Abingdon, UK) was used at 1/400. The mitochondrial biogenesis was evaluated by using rabbit polyclonal antibodies detecting AMP-activated protein kinase α (AMPKα)1/α2, defined as AMPKα (Cell signaling, ref: #2532), Sirtuin 1 (SIRT1) (Cell signaling, ref: #9475) and proliferator-activated receptor gamma co-activator-1 alpha (PGC-1α) (ref: ab54481; Abcam, Paris, France) and used at 1/1000. For studying the peroxisomal β-oxidation, the rabbit polyclonal anti-ABCD1 (serum 1664, Prof. P. Aubourg, Paris, France [71]) and anti-multifunctional enzyme type 2 (MFP2) (Gentex/Euromedex, Souffelweyersheim, France; #GTX114978) were used at a final dilution of 1/1000. An antibody directed against β-actin (mouse monoclonal antibody; ref: A2228; Sigma-Aldrich) was used at a final concentration of 1/10,000. After three 10 min washes with PBST, the membranes were incubated for 1 h at room temperature with a secondary horseradish peroxidase-conjugated goat anti-rabbit antibody (Cell Signaling, ref: #7074) or an anti-mouse antibody (Santa-Cruz Biotechnology, ref: #sc-2005) diluted at 1/5000 in 1% milk powder in PBST (PBS, 0.1% Tween 20, pH 7.2). The membranes were then washed, and antibody binding revealed using an enhanced chemiluminescence detection kit (Supersignal West Femto Maximum Sensitivity Substrate, Thermo Fisher Scientific) and Chemidoc XRS+ (Bio-Rad, Marnes la Coquette, France). The level of cleaved caspase-3, cleaved-PARP, Nrf2, GPx1, SOD1, SOD2, catalase, AMPKα, SIRT1, PGC-1α, ABCD1 and MFP2 were determined versus β-actin, and the (LC3-II/LC3-I) ratio were calculated with Image Lab software (Bio-Rad).

### 2.16. Real-Time Quantitative PCR Analysis

Total mRNA obtained from N2a cells after 48 h of treatment with RSV, QCT and API at 3.125 µM without or with 7KC (50 µM) were extracted and purified using the RNeasy Mini Kit (Qiagen, Courtaboeuf, France). Total mRNA concentration was measured with TrayCell (Hellma, Paris, France) by spectrophotometry (UV-1800, Shimadzu, Kyoto, Japan) at an absorbance of 260 nm and calculated with UV Probe (Shimadzu software, Shimadzu France, Marne la Vallée, France). The purity of nucleic acids was controlled by the ratio of absorbance at 260 nm and 280 nm (ratios of 1.8–2.2 were considered satisfactory). Then, 1 µg of total mRNA from each sample was converted into single-stranded cDNA using the iScript cDNA Synthesis kit (BioRad, Marne la Coquette, France) according to the following protocol: 5 min at 25 °C, 20 min at 46 °C, and 5 min at 95 °C. cDNA was then amplified in the presence of Takyon ^TM^ Rox SYBR Master Mix dTTP Blue (Eurogentec, Liége, Belgium) and 300 nM of forward and reverse mouse primers (Eurogentec, Liége, Belgium). The primer sequences were the following:

- Abcd1: forward 5′-gccaagttgtggatgtggag and reverse 5′-ttccgcagagtcgggataga-3′
- Abcd3: forward 5′-ggctgggcgtgaaatgacta-3′ and reverse 5′-gccgtttggaccacaaatca-3′
- Acox1: forward 5′-gcccaactgtgacttccatt-3′ and reverse 5′-ggcatgtaacccgtagcact-3′
- Ampkα1: forward 5′-catggctgagaagcagaagcac-3′ and reverse 5′-cttaactgccactttatggcctg-3′
- Gpx1: forward 5′-ccaccgtgtatgccttctcc-3′ and reverse 5′-agagagacgcgacattctcaat-3′
- Mfp2: forward 5′-aggggacttcaagggaattgg-3′ and reverse 5′-gcctgcttcaactgaatcgtaa-3′
- Nrf2: forward 5′-cagcatgttacgtgatgagg-3′ and reverse 5′-gctcagaaaaggctccatcc-3′
- Pex13: forward 5′-aaccaacacttacaagagtgcc-3′ and reverse 5′-ccgtaggctccatatccagaag-3′
- Pex14: forward 5′-acagcagtgaagttcctacaga-3′ and reevrse 5′-gccaggtcaatctcttcgtct-3′
- Pgc-1α: forward 5′-tgttcccgatcaccatattcc-3′ and reverse 5′-ggtgtctgtagtggcttgattc-3′
- Sod1: forward 5′-aaccagttgtgttgtcaggac-3′ and reverse 5′-ccaccatgtttcttagagtgagg-3′
- Sod2:forward 5′-cagacctgccttacgactatgg-3′ and reverse 5′-ctcggtggcgttgagattgtt-3′
- Sirt1: forward 5′-ttgtgaagctgttcgtggag-3′ and reverse 5′-ggcgtggaggtttttcagta-3′
- Thiolase A: forward 5′cctgaacagtgctgaagtgag-3′ and reverse 5′-acagtacacatttactgcatccc-3′

The real-time quantitative PCR products of reverse-transcribed cDNA samples were detected by StepOne Plus (Life Technologies/Thermo Fischer Scientific, Courtaboeuf, France). Thermal cycling conditions were as follows: activation of DNA polymerase at 95 °C for 10 min, followed by 40 cycles of amplification at 95 °C for 15 s, 60 °C for 30 s, and 72 °C for 30 s, followed by a melting curve analysis to test for the absence of non-specific products. Gene expression was quantified using cycle to threshold (Ct) values and normalized by the 36B4 reference gene (forward 5′-gcgacctggaagtccaacta-3′ and reverse 5′-atctgcttggagcccacat-3′). Specific amplification efficiencies were calculated with StepOne software (Life Technologies/Thermo Fischer Scientific). The quantitative expression of the studied genes was determined as fold induction of the control.

### 2.17. Statistical Analysis

Statistical analyses were perfomed using XLSTAT software (Microsoft). Data were expressed as mean ± standard deviation (SD); data were considered statistically different (Mann–Whitney test) at a *p*-value of 0.05 or less.

## 3. Results

### 3.1. Effects of Resveratrol, Quercetin, Apigenin and 7-Ketocholesterol on Cell Viability and Cell Growth Evaluated with the Fluoresceine Diacetate and Dulforhodamine 101 Assays

**Figure 1:**
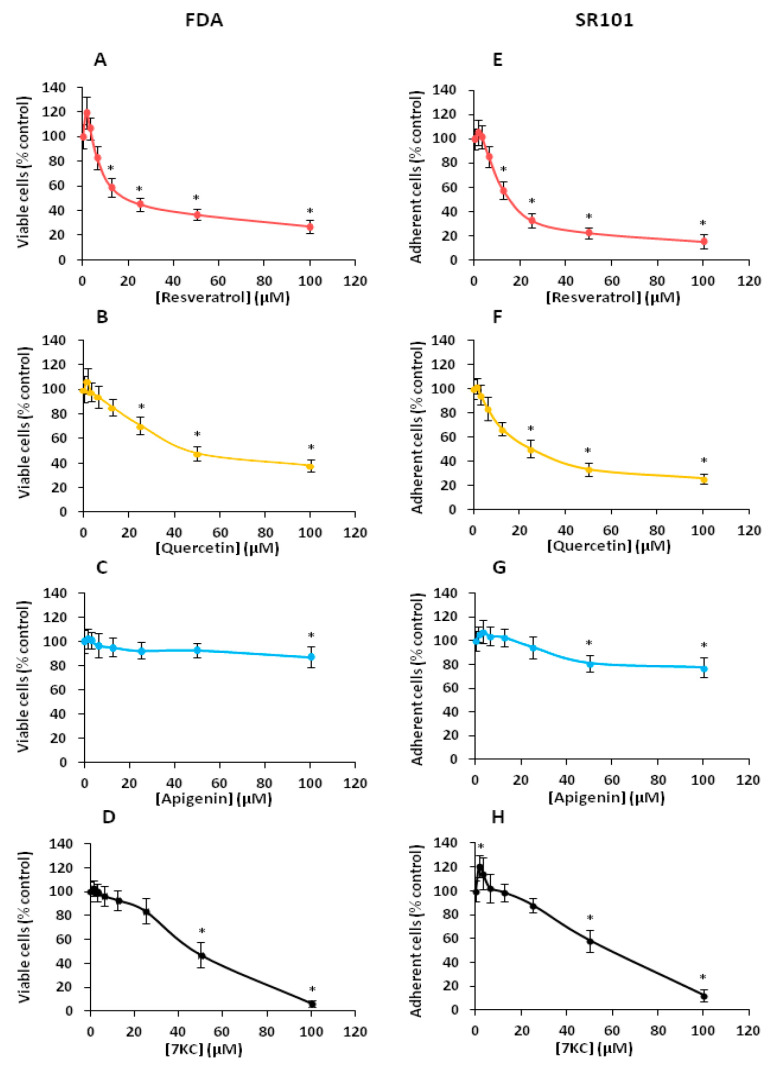
Effects of resveratrol, quercetin, apigenin and 7-ketocholesterol on cell viability and cell growth determined with the fluorescein diacetate and sulforhodamine 101 assays. Murine neuro-2a (N2a) neuroblastoma cells were cultured for 48 h with or without resveratrol (RSV), quercetin (QCT), apigenin (API) or 7-ketocholesterol (7KC; 1.5625 to 100 µM). The results are in percentages relative to the control (untreated cells). Data obtained with the fluorescein diacetate (FDA) assay (**A**–**D**), and the sulforhodamine (SR101) assay (**E**–**H**) are shown. Data shown are expressed as mean ± standard deviation (SD) of four independent experiments performed in triplicate. Significance of the differences between control (untreated cells) and RSV-, QCT-, API- and 7KC-treated cells; Mann–Whitney test: * *p* < 0.05 or less.

Whereas the polyphenols are known for their anti-oxidant properties, it is well known that some of them can have cytotoxic activities which depends on the concentration used [76]. Consequently, before simultaneously treating N2a cells in combination with 7KC and RSV, QCT or API for 48 h, we determined the cytotoxicity of these polyphenols. To this end, two assays were choosen: the fluorescein diacetate (FDA) assay, which is a widely used assay of cytotoxicity based on the measurement of esterase activity [16,17,70], and the sulforhodamine 101 (SR101) assay which permits the quantification of adherent cells to evaluate cell growth [16]. These two tests were performed because they give complementary information and can be carried out in microplates, allowing easy determination of the 50% inhibiting concentration (IC50) values. Compared to untreated cells, and in agreement with previous data [16,70], significant cytotoxic effects were observed with RSV (Figure 1A,E) and QCT (Figure 1B,F) from 12.5 to 100 µM, whereas slight cytotoxic effects were observed with API at 50 and 100 µM (Figure 1C,G). With 7KC (1.5625 to 100 µM), the IC50 was around 50 µM, both with the FDA and the SR101 assay (Figure 1D–H).

Thus, for further experiments, 7KC was used at 50 µM. So, among the concentrations of polyphenols chosen to assess cytoprotection, concentrations below 12.5 µM were used to avoid cytotoxicity (Figure 1). Of note, measurement of RSV, QCT and API uptake in N2a cells taking into account the fluorescence characteristics of these molecules showed concentration-dependent uptake (in the concentration range 3.125 to 25 µM) at culture times 3, 24 and 48 h (Figure S1).

### 3.2. Effects of Resveratrol, Quercetin, Apigenin and α-Tocopherol on 7-Ketocholesterol-Induced Plasma Membrane Damage Evaluated by Staining with Propidium Iodide

The effects of RSV, QCT and API on 7KC-induced plasma membrane damage was evaluated with propridium iodide (PI). A wide range of polyphenol concentrations was used (1.5 to 25 µM) in order to precisely identify the concentrations capable of preventing plasma membrane damages (increased permeability to PI). PI was chosen to evaluate plasma membrane permeability, due to its ability to enter into cells with damaged and permeable plasma membranes, considered as dead cells [80].

**Figure 2:**
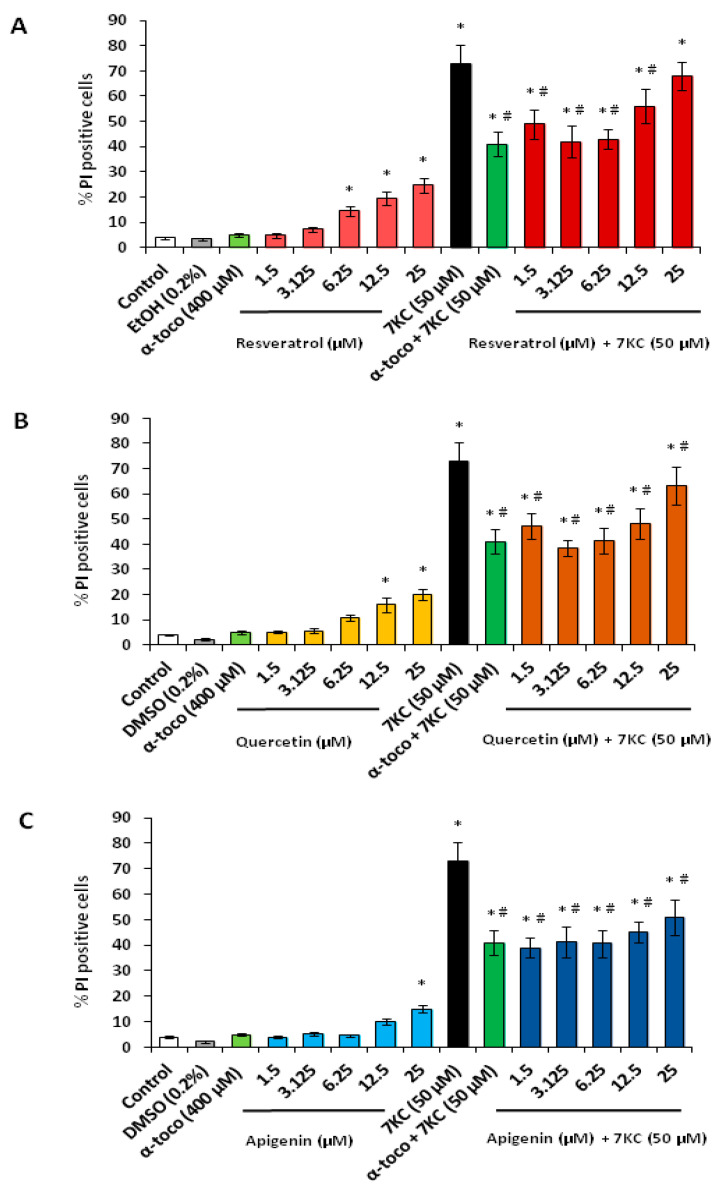
Effects of resveratrol, quercetin, apigenin or α-tocopherol on 7-ketocholesterol-induced plasma membrane permeability evaluated with propidium iodide. N2a cells were cultured for 48 h with or without 7-ketocholesterol (7KC, 50 μM) in the presence or absence of α-tocopherol (α-toco: 400 µM) used as the positive control for cytoprotection, or with polyphenols: resveratrol (RSV) (**A**), quercetin (QCT) (**B**) or apigenin (API) (**C**) at concentrations ranging from 1.5625 (written 1.5) to 26 µM. Plasma membrane permeability was measured by flow cytometry with propidium iodide (PI): for each assay, the percentage of PI positive cells was determined. Two vehicle controls were realized: Ethanol (EtOH) (0.2%) used with RSV and 7KC, and DMSO (0.2%) used with QCT and API. Each value is the mean ± standard deviation (SD) of four independent experiments. Significance of the differences between control (untreated cells) and RSV-, QCT-, API-, α-toco- or 7KC-treated cells; Mann–Whitney test: * *p* < 0.05 or less. Significance of the differences between 7KC-treated cells and (7KC + (RSV, QCT, API or α-toco))-treated cells; Mann–Whitney test: # *p* < 0.05 or less. No significant differences were found between control and vehicle-treated cells (Ethanol (EtOH): 0.2% and DMSO: 0.2%).

With API, a slight but significant increase in PI positive cells was observed only at 25 µM (Figure 2C). However, in the presence of 7KC (50 µM, 48 h), the percentage of PI positive cells was strongly increased (Figure 2A–C). When 7KC was simultaneously incubated with RSV, QCT and API, the increase in PI positive cells was significantly attenuated at concentrations from 1.5 to 25 µM: the percentages of PI positive cells were reduced compared to 7KC-treated cells (Figure 2A–C). The most pronounced effects were observed at 3.125 and 6.25 µM, with RSV and QCT (Figure 2A,B). With API, similar cytoprotective effects were observed from 1.5 to 25 µM (Figure 2C). With α-tocopherol (400 µM), used as a reference to prevent 7KC-induced side effects, cytoprotective effects were found with the PI assay (Figure 2).

Consistent with the results obtained on other adherent cells treated with 7KC, the increased permeability of the plasma membrane to PI was associated with a loss of cell adhesion [81,82]. Thus, an increase in round cells suspended in the culture medium, considered as dead cells, was observed (Figure S2). Cytoprotection associated with RSV, QCT and API, as well as by α-tocopherol, was accompanied by a restoration of cell adhesion. No marked morphological differences were detected between untreated cells (control), cells treated with vehicles (ethanol or DMSO) and cells treated with RSV, QCT, API and α-tocopherol. However, in the presence of the polyphenols and at the low concentrations used, some cells with dendrites that may result from the differentiating activity of the polyphenols [15,16] were sometimes observed.

To ensure that the cytoprotection against 7KC observed with polyphenols (RSV, QCT, API) was not species and neuronal cell type dependent, human SK-N-BE and SH-SY5Y nerve cells were used. In SK-N-BE and SH-SY5Y cells, under co-treatment conditions and using the FDA test, cytoprotection was also observed with RSV, QCT and API (Figures S3 and S4). The most effective cytoprotective concentrations were usually 3.125, 6.25 and/or 12.5 µM (Figures S3 and S4).

In addition, when cells were pre-treated with polyphenols, prior to the addition of 7KC, equivalent and sometimes higher cytoprotection were observed than in co-treatment (Figure S5). On the other hand, in post-treatment, the cytoprotection observed was always lower than in co-treatment and pre-treatment (Figure S5). The subsequent results presented were obtained on N2a cells under co-treatment conditions.

### 3.3. Effects of Resveratrol, Quercetin, Apigenin and α-Tocopherol on 7-Ketocholesterol-Induced Oxidative Stress

**Figure 3:**
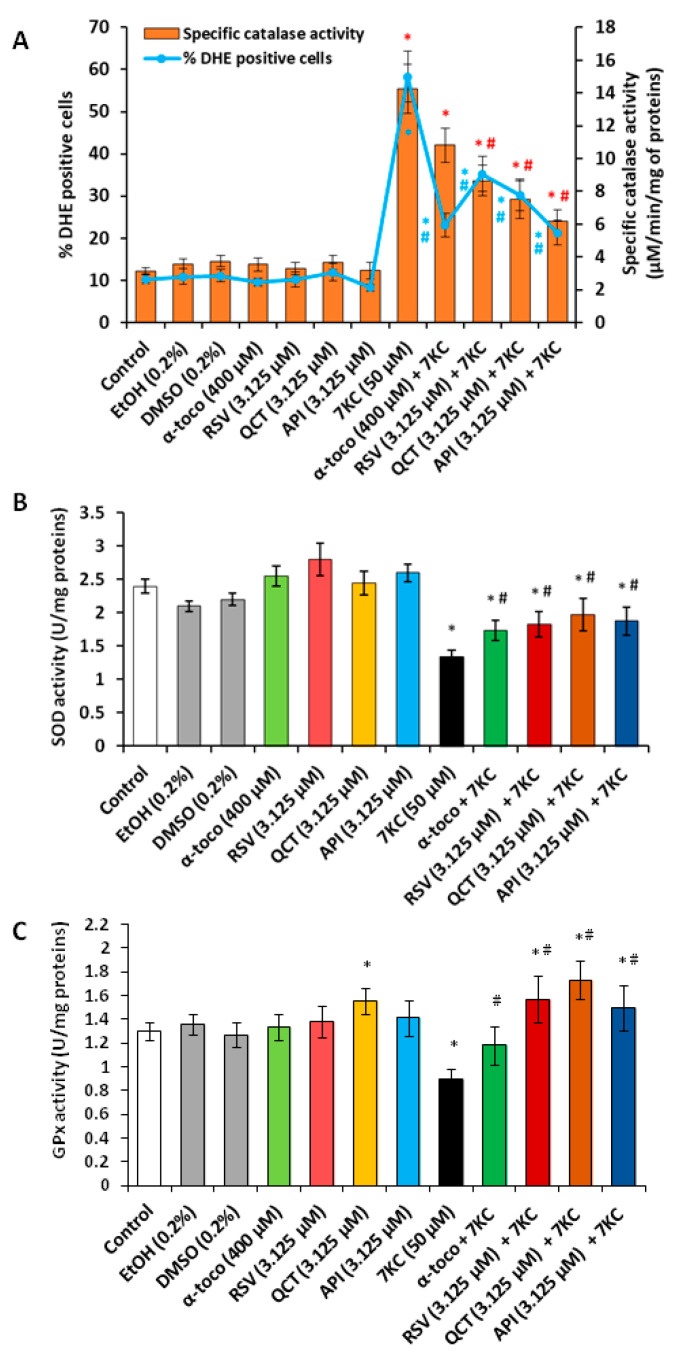
Effect of resveratrol, quercetin, apigenin and α-tocopherol on 7-ketocholesterol-induced oxidative stress: ROS overproduction and catalase activity, measurement of superoxide dismutase and glutathione peroxidase activity. N2a cells were cultured for 48 h with or without 7-ketocholesterol (7KC, 50 μM) in the presence or absence of α-tocopherol (α-toco: 400 µM) used as a positive control for cytoprotection, or with polyphenols: resveratrol (RSV), quercetin (QCT) or apigenin (API) used at a concentration of 3.125 and/or 6.25 µM. (**A**) ROS overproduction was measured by flow cytometry after staining with dihydroethidine (DHE) and evaluated by the percentage of DHE positive cells. The effect on catalase activity, a peroxisomal antioxidant enzyme, which degrades hydrogen peroxide (H_2_O_2_), was determined by a colorimetric assay with the measurement of H_2_O_2_ consumption. The enzymatic activities of superoxide dismutase (SOD) (**B**) and glutathione peroxidase (GPx) (**C**) were determined by colorimetric assays. Data shown are mean ± standard deviation (SD) of three independent experiments conducted in triplicate. Significance of the differences between control (untreated cells) and RSV-, QCT-, API-, α-toco- or 7KC-treated cells; Mann–Whitney test: * *p* < 0.05 or less. Significance of the differences between 7KC-treated cells and (7KC + (RSV, QCT, API or α-toco)- treated cells; Mann–Whitney test: # *p* < 0.05 or less. No significant differences were found between control and vehicle-treated cells (Ethanol (EtOH): 0.2% and DMSO: 0.2%).

The effect of 7KC (50 µM) in the presence or absence of 3.125 µM RSV, QCT or API on oxidative stress was determined after 48 h of treatment, both by flow cytometry with dihydrohetidine (DHE), by measuring catalase, SOD and GPx activities, by real time-quantitative polymerase chain reaction (RT-qPCR) and by Western blot to quantify the mRNA and the protein levels of anti-oxidant enzymes (GPx1: glutathione peroxidase 1 localized in the cytosol is a selenoenzyme involved in the detoxification of hydrogen peroxide (H_2_O_2_); SOD1: superoxide dismutase 1 localized in the cytosol is also named CuZn SOD and involved in the detoxification of superoxide anion (O_2_^•−^); SOD2: superoxide dismutase 2 localized in the mitochondria is also named Mn SOD and involved in the detoxification of O_2_^•−^). Catalase is a peroxisomal enzyme which is increased in oxidative stress conditions and which allows the dismutation of hydrogen peroxide in water and dioxygen (2 H_2_O_2_ → 2 H_2_O + O_2_). We also measured the mRNA and protein level of the transcription factor Nrf2 (nuclear factor erythroid-2-related factor 2) which activates the expression of numerous genes encoding for enzymes involved in the prevention of oxidative stress such as quinine oxidoreductase (NQO1), hemeoxigenase 1 (HMOX1) and glutathione peroxidase (GPx) [83]. Under our experimental conditions, 7KC induced a marked ROS overproduction (increased percentage of DHE positive cells), which was strongly counteracted by RSV, QCT and API; API was the most efficient compared to RSV and QCT, but also relative to α-tocopherol (400 µM) used as positive cytoprotective molecule (Figure 3A). 7KC also simultaneously induced an important increase in catalase activity; this increase was reduced by RSV, QCT and API; API was the most efficient compared with RSV and QCT, but also to α-tocopherol used as positive cytoprotective molecule (Figure 3A). RSV, QCT and API as well as α-tocopherol, used alone, have no or slight effects on ROS production and catalase activity (Figure 3A).

**Figure 4:**
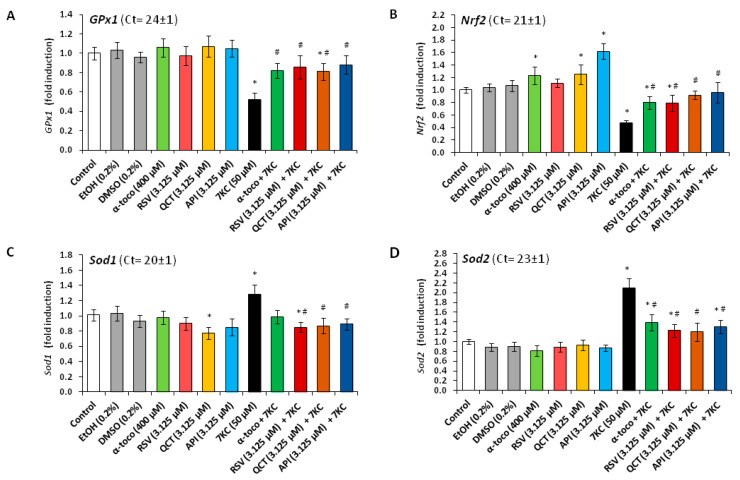
Effect of resveratrol, quercetin, apigenin or α-tocopherol on 7KC-induced oxidative stress: measurement of *GPx1*, *Nrf2*, *Sod1* and *Sod2* mRNA levels by real time-quantitative polymerase chain reaction. N2a cells were cultured for 48 h with or without 7-ketocholesterol (7KC, 50 μM) in the presence or absence of α-tocopherol (α-toco: 400 µM) used as a positive control for cytoprotection, or with polyphenols: resveratrol (RSV), quercetin (QCT) or apigenin (API) used at a concentration of 3.125 µM. The relative expression of *GPx1* (**A**), *Nrf2* (**B**), *Sod1* (**C**) and *Sod2* (**D**) mRNAs was determined by real time-quantitative polymerase chain reaction (RT-qPCR). In untreated cells, the cycle threshold (Ct) values are provided for each gene studied: *GPx1* (Ct = 24 ± 1), *Nrf2* (Ct = 21 ± 1), *Sod1* (Ct = 20± 1) and *Sod2* (Ct = 23 ± 1). Data shown are mean ± standard deviation (SD) of two independent experiments conducted in triplicate. Significance of the differences between control (untreated cells) and RSV-, QCT-, API-, α-toco- or 7KC-treated cells; Mann–Whitney test: * *p* < 0.05 or less. Significance of the differences between 7KC-treated cells and (7KC + (RSV, QCT, API or α-toco))-treated cells; Mann–Whitney test: # *p* < 0.05 or less. No significant differences were found between control and vehicle-treated cells (Ethanol (EtOH): 0.2% and DMSO: 0.2%).

**Figure 5:**
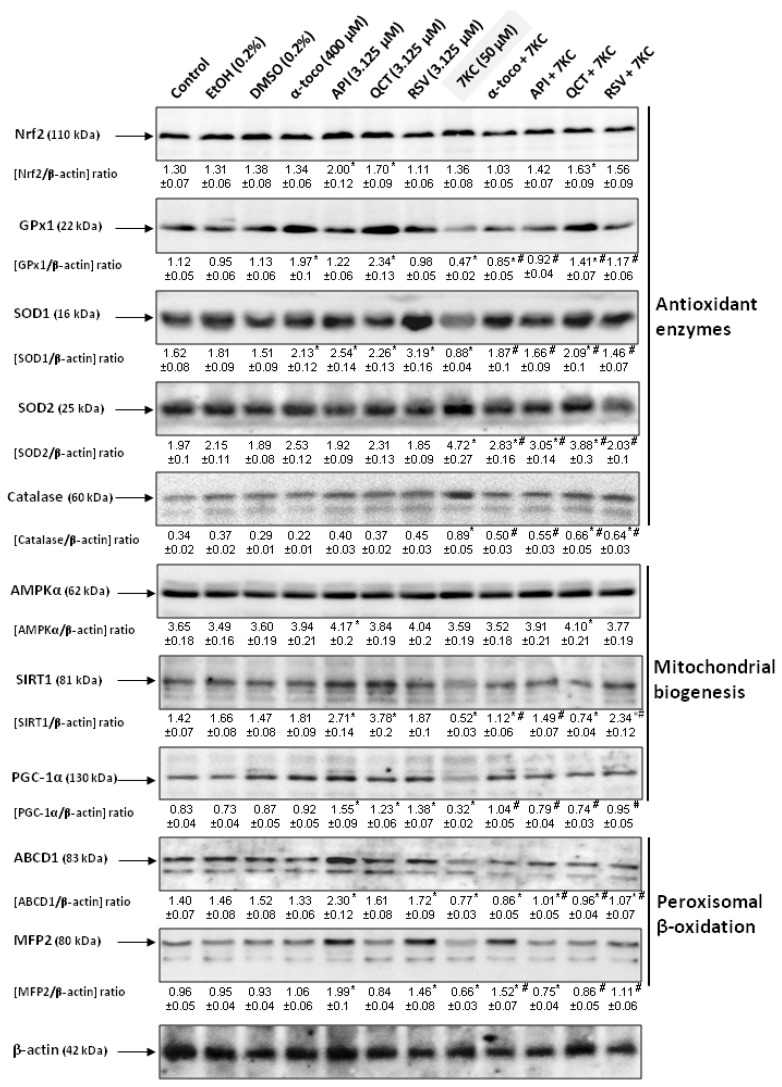
Effects of resveratrol, quercetin, apigenin, α-tocopherol and 7-ketocholesterol on the expression of antioxidant proteins (Nrf2, GPx1, SOD1, SOD2, catalase), mitochondrial biogenesis proteins (AMPKα, SIRT1, PGC-1α) and enzymes involved in the peroxisomal β-oxidation (ABCD1, MFP2) in murine N2a neuroblastoma cells. N2a cells previously cultured for 24 h were cultured for 48 h with or without 7-ketocholesterol (7KC, 50 μM) in the presence or absence of polyphenols: resveratrol (RSV), quercetin (QCT) or apigenin (API) at 3.125 µM or α-tocopherol (400 µM). Representative Western blots for analysis were presented, showing protein abundance of antioxidant protein markers (expression of nuclear factor erythroid 2-related factor 2 (Nrf2), glutathione peroxidase (GPx1), superoxide dismutase 1 (SOD1), superoxide dismutase 2 (SOD2) and catalase), mitochondrial biogenesis proteins (AMP-activated protein kinase- alpha (AMPKα), Sirtuin 1 (SIRT1) and peroxisome proliferator-activated receptor gamma co-activator-1 alpha (PGC-1α)) and of proteins involved in peroxisomal β-oxidation (ATP binding cassette subfamily D member 1 (ABCD1) and peroxisomal multifunctional enzyme type 2 (MFP2). β-actin was used as the loading control. The intensities of the bands for each set were individually determined and are presented as the ratio over β-actin signal. The EtOH value (0.2%) and the DMSO value (0.2%) correspond to the final EtOH and DMSO concentration in the culture medium. No difference was observed between control and vehicle (EtOH and DMSO)-treated cells. Data shown are representative of three independent experiments. Significance of the differences between control (untreated cells) and RSV-, QCT-, API-, α-toco- or 7KC-treated cells; Mann–Whitney test: * *p* < 0.05 or less. Significance of the differences between 7KC-treated cells and (7KC + (RSV, QCT, API or α-toco))-treated cells; Mann–Whitney test: # *p* < 0.05 or less. No significant differences were found between control and vehicle-treated cells (Ethanol (EtOH): 0.2% and DMSO: 0.2%).

Measurement of enzyme activities also showed a decrease in GPx and SOD activities in the presence of 7KC; the decrease in these activities was strongly and significantly attenuated in the presence of RSV, QCT, API and α-tocopherol, without returning to levels similar to those of control and vehicle-treated cells (Figure 3B,C). In the presence of 7KC, compared to control cells, the relative expression of *Gpx1* and *Nrf2* mRNAs were strongly reduced (Figure 4A,B), whereas the relative expression of *Sod1* and *Sod2* was increased (Figure 4C,D). No significant differences were observed between control and vehicle-treated cells (EtOH (0.2%) and DMSO (0.2%) (Figure 4A–D). When associated with 7KC, the polyphenols (RSV, QCT and API), as well as α-tocopherol, tended to normalize the expression of *GPx1*, *Nrf2*, *Sod1* and *Sod2* (Figure 4A–D). These data demonstrate the benefit of RSV, QCT, API and α-tocopherol on *GPx1*, *Nrf2*, *Sod1* and *Sod2* gene expression. Compared to control cells, RSV used alone has no effect on the mRNA levels of *GPx1*, *Nrf2*, *Sod1* and *Sod2* (Figure 4A–D), whereas QCT and API increased the *Nrf2* mRNA level (Figure 4B). With α-tocopherol, the *Nrf2* mRNA level was also enhanced (Figure 4B). On the other hand, the measurement of the amount of GPx1, SOD1, SOD2 and catalase proteins by Western blot revealed decreases in the expression of GPx1 and SOD1 and increases in SOD2 and catalase in the presence of 7KC (Figure 5). When RSV, QCT and API as well as α-tocopherol were associated with 7KC, these changes were strongly and significantly attenuated, and the values observed were close to those observed in control and vehicle-treated cells (Figure 5). Altogether, these data show that 7KC induces oxidative stress on N2a cells, and that this effect is prevented by RSV, QCT, API and α-tocopherol.

### 3.4. Effects of Resveratrol, Quercetin and Apigenin on 7-Ketocholesterol-Induced Mitochondrial Dysfunction

**Figure 6:**
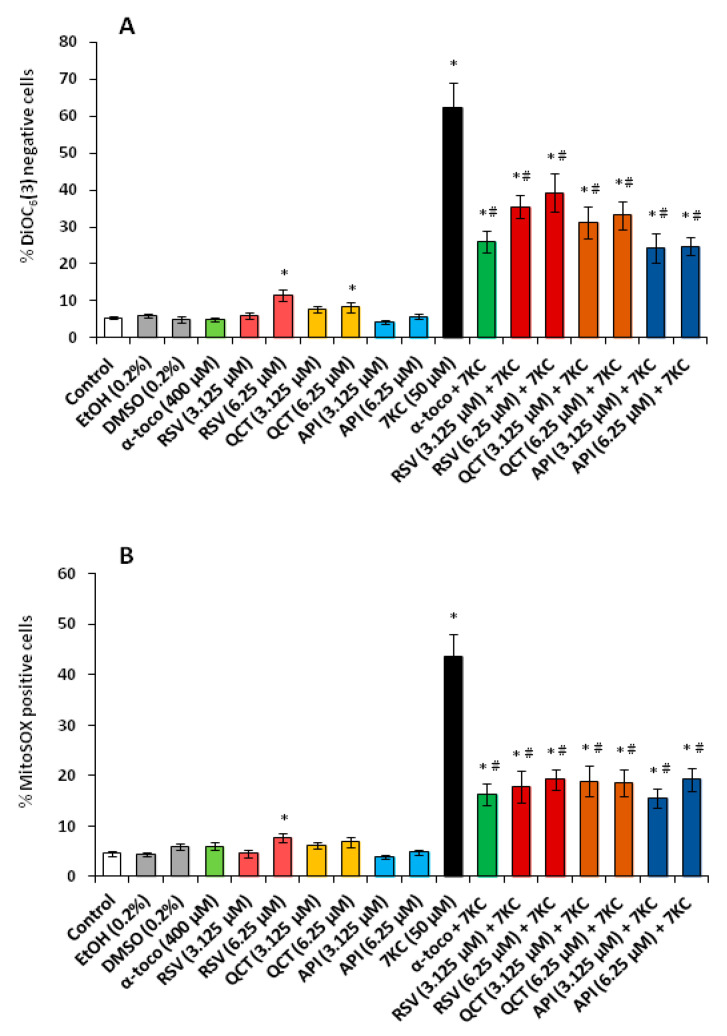
Effects of resveratrol, quercetin, apigenin and α-tocopherol on 7-ketocholesterol-induced mitochondrial dysfunction: loss of mitochondrial membrane potential measured with DiOC_6_(3) and mitochondrial ROS overproduction measured with MitoSOX. N2a cells were cultured for 48 h with or without 7-ketocholesterol (7KC, 50 μM) in the presence or absence of α-tocopherol (α-toco: 400 µM) used as a positive control for cytoprotection, or with polyphenols: resveratrol (RSV), quercetin (QCT) or apigenin (API) used at concentrations of 3.125 and 6.25 µM. Loss of mitochondrial membrane potential (ΔΨm) was measured by flow cytometry after staining with DiOC_6_(3) and evaluated by the percentage of DiOC_6_(3) negative cells (**A**). The effect on ROS overproduction at the mitochondrial level was determined by flow cytometry after staining with MitoSOX and evaluated by the percentage of MitoSOX positive cells (**B**). Data shown are mean ± standard deviation (SD) of three independent experiments conducted in triplicate. Significance of the differences between control (untreated cells) and RSV-, QCT-, API-, α-toco- or 7KC-treated cells; Mann–Whitney test: * *p* < 0.05 or less. Significance of the differences between 7KC-treated cells and (7KC + (RSV, QCT, API or α-toco))-treated cells; Mann–Whitney test: # *p* < 0.05 or less. No significant differences were found between control and vehicle-treated cells (Ethanol (EtOH): 0.2% and DMSO: 0.2%).

DiOC_6_(3) and MitoSOX are widely used to study the drop of mitochondrial membrane potential (ΔΨm) and ROS overproduction at the mitochondrial level, respectively. Under treatment with 7KC (50 µM, 48 h), an important increase in N2a cells with depolarized mitochondria (percentages of DiOC_6_(3) negative cells) was observed, as well as an increase in cells characterized by an overproduction of ROS at the mitochondrial level (percentage of MitoSOX positive cells) (Figure 6A,B).

**Figure 7:**
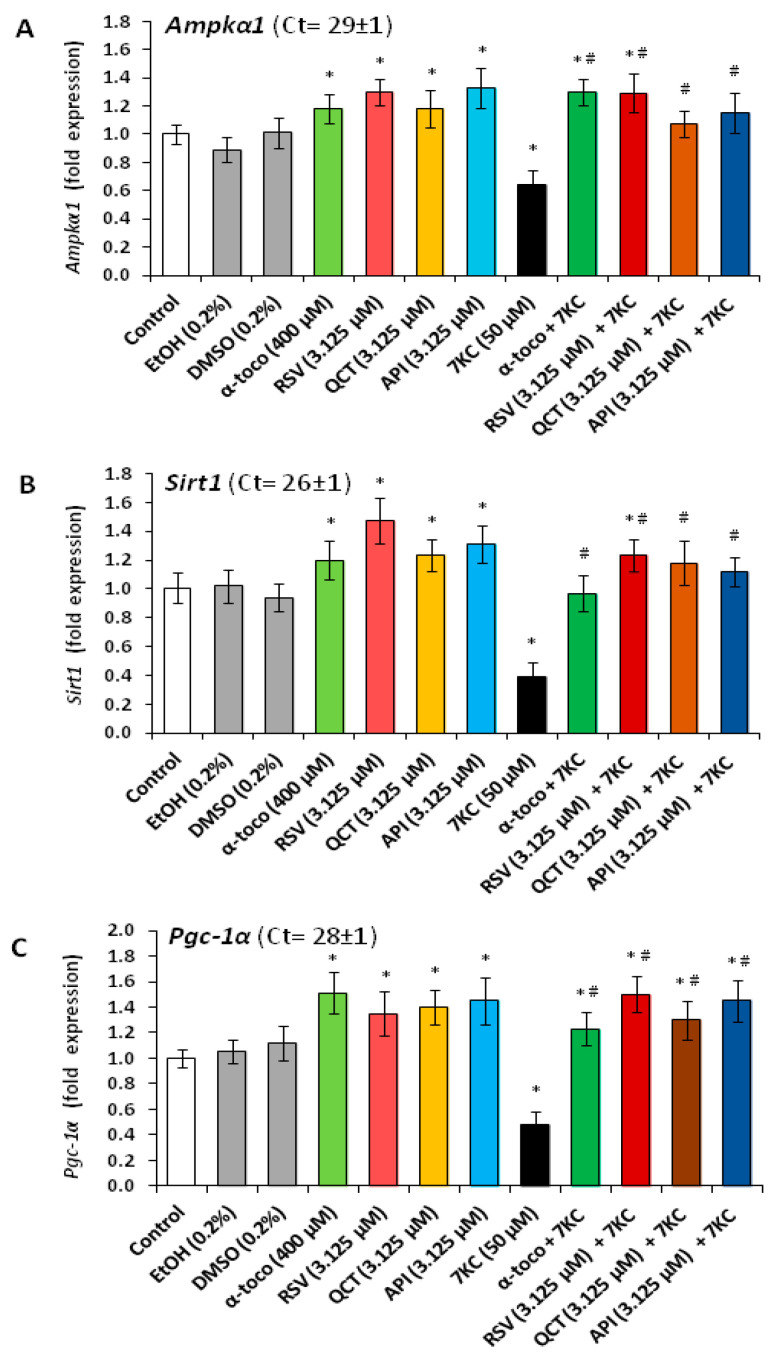
Effect of resveratrol, quercetin, apigenin or α-tocopherol on 7KC-induced mitochondrial biogenesis disorders: measurement of the mRNA levels of Ampkα1, Sirt1 and Pgc-1α by real time-quantitative polymerase chain reaction. N2a cells were cultured for 48 h with or without 7-ketocholesterol (7KC, 50 μM) in the presence or absence of α-tocopherol (α-toco: 400 µM) used as a positive control for cytoprotection, or with polyphenols: resveratrol (RSV), quercetine (QCT) or apigenin (API) used at a concentration of 3.125 µM. The relative expression of Ampkα1 (**A**), Sirt1 (**B**), and Pgc-1α (**C**) mRNAs was determined by real time-quantitative polymerase chain reaction (RT-qPCR). In untreated cells, the cycle threshold (Ct) values are provided for each gene studied: *Ampkα1* (Ct = 29 ± 1), *Sirt1* (Ct = 26 ± 1), and *Pgc-1α* (Ct = 28 ± 1). Data shown are mean ± standard deviation (SD) of two independent experiments conducted in triplicate. Significance of the differences between control (untreated cells) and RSV-, QCT-, API-, α-toco- or 7KC-treated cells; Mann–Whitney test: * *p* < 0.05 or less. Significance of the differences between 7KC-treated cells and (7KC + (RSV, QCT, API or α-toco))-treated cells; Mann–Whitney test: # *p* < 0.05 or less. No significant differences were found between control and vehicle-treated cells (Ethanol (EtOH): 0.2% and DMSO: 0.2%).

Notably, the loss of ΔΨm was prevented by co-treatment with RSV, QCT or API used at 3.125 and 6.25 µM (API being the most efficient) and by α-tocopherol (400 µM) (Figure 6A). The overproduction of ROS at the mitochondrial level was also strongly and equally reduced by RSV, QCT, API and α-tocopherol (Figure 6B). By RT-qPCR, under treatment with 7KC, we also observed an important reduction in the mRNA levels of *Ampkα1*, *Sirt1* and *Pgc-1α* encoding for proteins which favor mitochondrial biogenesis [84] (Figure 7A–C).

When N2a cells were cultured with 7KC associated with RSV, QCT or API, or with α-tocopherol, the decrease in the mRNA levels was counteracted, and the mRNAs levels of *Ampkα1*, *Sirt1* and *Pgc-1α* were similar to those in in untreated N2a cells (Figure 7A–C). Western blot measurement of the amount of protein AMPKα, SIRT1 and PGC-1α revealed decreases in the level of SIRT1 and PGC-1α in the presence of 7KC whereas AMPKα level was unchanged (Figure 5). When RSV, QCT and API or α-tocopherol were associated with 7KC, these changes were strongly and significantly attenuated, and the values observed were close to those measured on control and vehicle-treated cells (Figure 5). Thus, the effects of 7KC at the mitochondrial level (loss of ΔΨm; mitochondrial ROS overproduction; reduction in the mRNA and/or protein levels of Ampkα, SIRT1 and PGC-1α) are prevented by RSV, QCT and API, as well as by α-tocopherol.

### 3.5. Effects of Resveratrol, Quercetin, Apigenin and α-Tocopherol on 7-Ketocholesterol-Induced Peroxisomal Dysfunction

**Figure 8:**
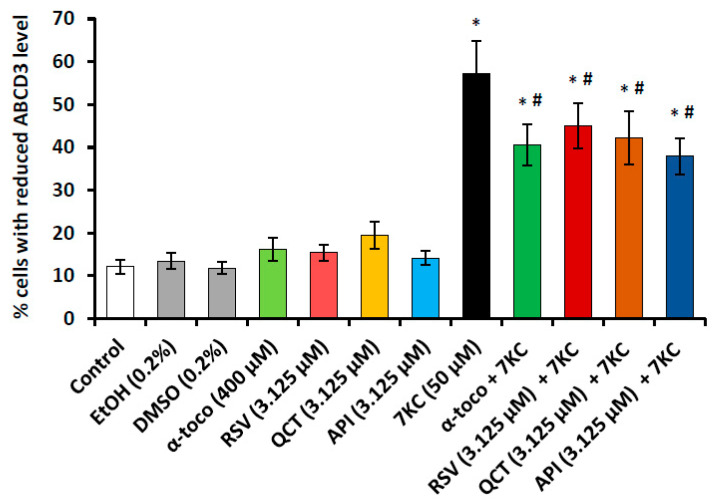
Flow cytometric evaluation of the effect of resveratrol, quercetin, apigenin or α-tocopherol on 7KC-induced changes of peroxisomal mass evaluated with ABCD3 expression. N2a cells were cultured for 48 h with or without 7-ketocholesterol (7KC, 50 μM) in the presence or absence of α-tocopherol (α-toco: 400 µM) used as a positive control for cytoprotection, or with polyphenols: resveratrol (RSV), quercetin (QCT) or apigenin (API) used at a concentration of 3.125 µM. The level of ABCD3 was evaluated by flow cytometry: the percentage of cells with lower ABCD3 levels, compared to untreated cells and vehicle-treated cells (EtOH 0.2%; DMSO 0.2%) was determined. Data shown are mean ± standard deviation (SD) of three independent experiments conducted in triplicate. Significance of the differences between control (untreated cells) and RSV-, QCT-, API-, α-toco- or 7KC-treated cells; Mann–Whitney test: * *p* < 0.05 or less. Significance of the differences between 7KC-treated cells and (7KC + (RSV, QCT, API or α-toco))-treated cells; Mann–Whitney test: # *p* < 0.05 or less. No significant differences were found between control and vehicle-treated cells (Ethanol (EtOH): 0.2% and DMSO: 0.2%).

The incidence of 7KC on the peroxisomal mass was evaluated with a rabbit polyclonal antibody raised against the peroxisomal ABCD3 transporter, which is a major protein of the peroxisomal membrane, as previously described [43,68]. The impact on the peroxisomal mass and biogenesis was also evaluated by RT-qPCR via the quantification of *Abcd3*, *Pex13* and *Pex14* mRNAs. The peroxines 13 and 14 (PEX13, PEX14) constitute a docking complex localized at the peroxisomal membrane level and are required for peroxisome biogenesis [85]. The negative impact on the peroxisomal β-oxidation, which could favor neurodegeneration [86], was also evaluated by RT-qPCR via the quantification of the mRNAs encoding the ABCD1 transporter, and of the enzymes acyl-CoA oxidase 1 (ACOX1), multifunctional protein-2 (MFP2) and thiolase A which are required for the β-oxidation of very long chain fatty acids (VLCFA) [87] (Figure S6). In agreement with previous data obtained on 158N and BV-2 cells [45,76], an increase in the percentage of cells with lower ABCD3 expression, suggesting a decrease in peroxisomal mass per cell, was observed (Figure 8). This increase was attenuated by RSV, QCT and API (Figure 8).

**Figure 9:**
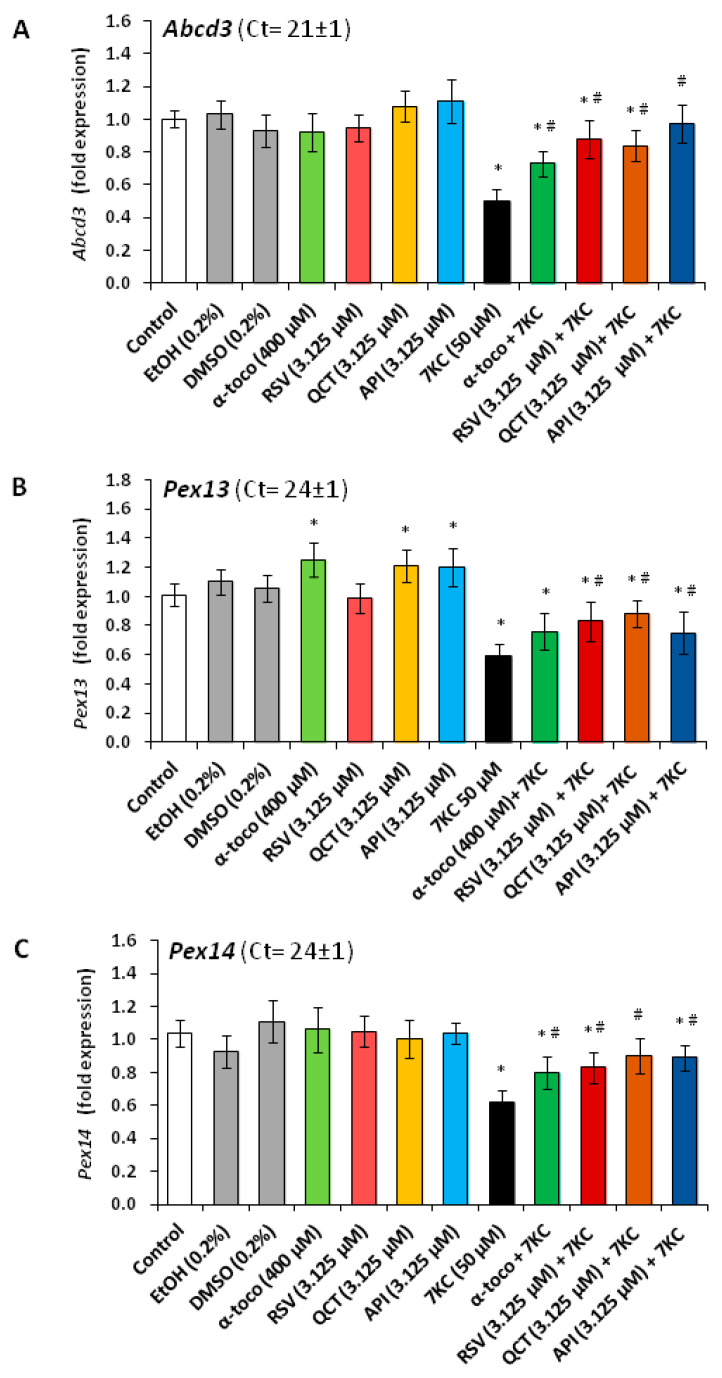
Effect of resveratrol, quercetin, apigenin or α-tocopherol on 7KC-induced changes of peroxisomal mass and biogenesis: measurement of the mRNA levels of *Abcd3*, *Pex13* and *Pex14* by real time-quantitative polymerase chain reaction. N2a cells were cultured for 48 h with or without 7-ketocholesterol (7KC, 50 μM) in the presence or absence of α-tocopherol (α-toco: 400 µM) used as a positive control for cytoprotection, or with polyphenols: resveratrol (RSV), quercetin (QCT) or apigenin (API) used at a concentration of 3.125 µM. The relative expression of *Abcd3* (**A**), *Pex13* (**B**), and *Pex14* (**C**) mRNAs was determined by real time-quantitative polymerase chain reaction (RT-qPCR). In untreated cells, the cycle threshold (Ct) values are provided for each gene studied: *Abcd3* (Ct = 21 ± 1), *Pex13* (Ct = 24 ± 1), and *Pex14* (Ct = 24 ± 1). Data shown are mean ± standard deviation (SD) of two independent experiments conducted in triplicate. Significance of the differences between control (untreated cells) and RSV-, QCT-, API-, α-toco- or 7KC-treated cells; Mann–Whitney test: * *p* < 0.05 or less. Significance of the differences between 7KC-treated cells and (7KC + (RSV, QCT, API or α-toco))-treated cells; Mann–Whitney test: # *p* < 0.05 or less. No significant differences were found between control and vehicle-treated cells (Ethanol (EtOH): 0.2% and DMSO: 0.2%).

**Figure 10:**
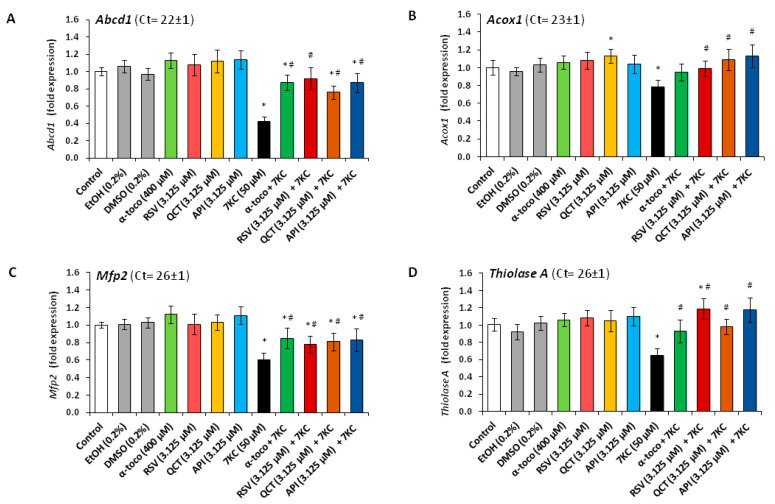
Effect of resveratrol, quercetin, apigenin and α-tocopherol on 7KC-induced changes of peroxisomal β-oxidation: measurement of the mRNA levels of *Abcd1*, *Acox1*, *Mfp2* and *Thiolase A* by real time-quantitative polymerase chain reaction. N2a cells were cultured for 48 h with or without 7-ketocholesterol (7KC, 50 μM) in the presence or absence of α-tocopherol (α-toco: 400 µM) used as a positive control for cytoprotection, or with polyphenols: resveratrol (RSV), quercetine (QCT) or apigenin (API) used at a concentration of 3.125 µM. Beta-Oxidation of very long chain fatty acids (VLCFAs) in peroxisomes: the implication of ABCD1, ACOX1, MFP2 and Thiolase A in peroxisomal β-oxidation is summarized in (**A**). The relative expression of *Abcd1* (**B**), *Acox1* (**C**), *Mfp2 (***D**) and *Thiolase A* (**E**) mRNAs was determined by real time-quantitative polymerase chain reaction (RT-qPCR). In untreated cells, the cycle threshold (Ct) values are provided for each gene studied: *Abcd1* (Ct = 22 ± 1), *Acox1* (Ct = 23 ± 1), *Mfp2* (Ct = 26 ± 1) and Thiolase A (Ct = 26 ± 1). Data shown are mean ± standard deviation (SD) of two independent experiments conducted in triplicate. Significance of the differences between control (untreated cells) and RSV-, QCT-, API-, α-toco- or 7KC-treated cells; Mann–Whitney test: * *p* < 0.05 or less. Significance of the differences between 7KC-treated cells and (7KC + (RSV, QCT, API or α-toco))-treated cells; Mann–Whitney test: # *p* < 0.05 or less. No significant differences were found between control and vehicle-treated cells (Ethanol (EtOH): 0.2% and DMSO: 0.2%).

In addition, as shown by RT-qPCR, the important decreases in *Abcd3*, *Pex13* and *Pex14* mRNA levels, observed under treatment with 7KC (50 µM, 48 h) were prevented by co-treatment with RSV, QCT or API (3.125 µM), as well as α-tocopherol (400 µM) (Figure 9). In addition, 7KC also induced a decreased gene expression (*Abcd1*, *Acox1*, *Mfp2* and *thiolase A*), which was significantly attenuated by co-treatment with RSV, QCT, API (3.125 µM) or α-tocopherol (400 µM) (Figure 10). By Western blot, decreases in the peroxisomal proteins ABCD1 and MFP2, associated with peroxisomal β-oxidation (Figure S6), were also found in the presence of 7KC (Figure 5). When RSV, QCT and API were associated with 7KC, these decreases were attenuated (Figure 5). Compared to 7KC, α-tocopherol associated with 7KC increased the expression of ABCD1 and MFP2 (Figure 5). These data show that 7KC-induced peroxisomal changes impacts peroxisomal mass, biogenesis and activity, and that these changes are attenuated by RSV, QCT, API or α-tocopherol.

### 3.6. Characteristics of 7-Ketocholesterol-Induced Cell Death in N2a Cells: Evidence of Apoptotic and Autophagic Criteria, and Determination of the Effects of Resveratrol, Quercetin, Apigenin or α-Tocopherol

**Figure 11:**
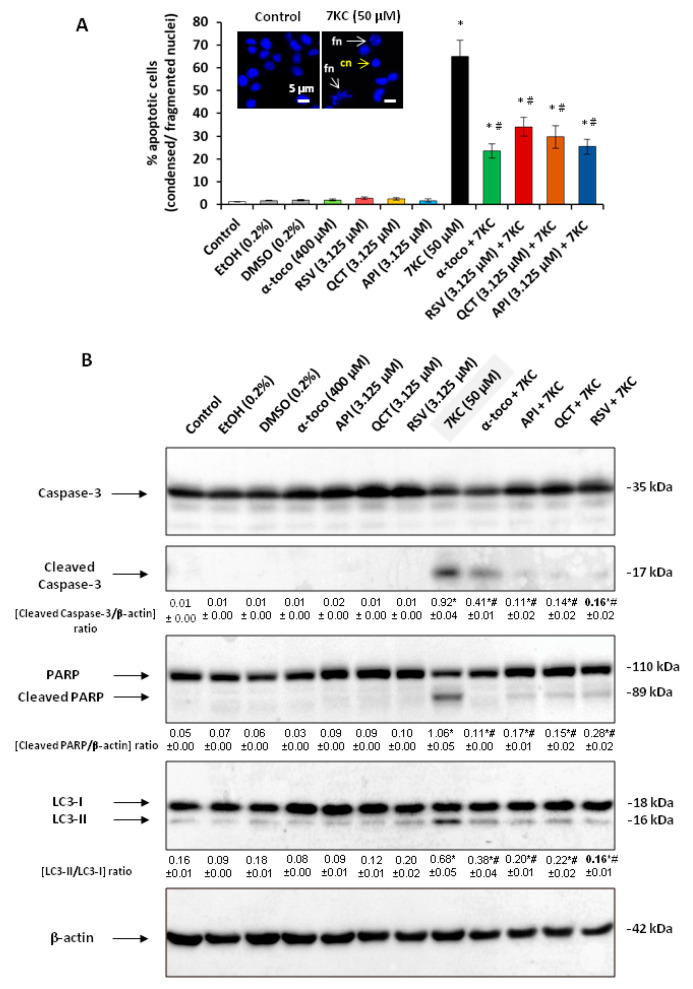
Effect of resveratrol, quercetin, apigenin and α-tocopherol on 7KC-induced autophagy and apoptosis: characterization of apoptotic nuclei by staining with Hoechst 33342, identification of cleaved-caspase-3 and PARP and activation of LC3-I into LC3-II by Western blotting. N2a cells were cultured with or without 7-ketocholesterol (7KC: 50 μM, 48 h) in the presence or absence of α-tocopherol (α-toco: 400 µM) used as a positive control for cytoprotection, or with polyphenols: resveratrol (RSV), quercetin (QCT) or apigenin (API) used at 3.125 µM. (**A**) Cells with condensed nuclei (cn) and/or fragmented nuclei (fn) characteristic of apoptotic cells are identified. When 7KC was associated with RSV, QCT and API, the presence of apoptotic cells was strongly reduced; no or few apoptotic cells were present in control (untreated cells), vehicle-treated cells (EtOH 0.2%; DMSO 0.2%) and RSV-, QCT- and API-treated cells. (**B**) Apoptosis was also evaluated by caspase-3 activation (cleaved caspase-3), and PARP fragmentation, and autophagy was evaluated by conversion of LC3-I to LC3-II (increased ratio (LC3-II / LC3-I)). The EtOH value (0.2%) and the DMSO value (0.2%) correspond to the final EtOH and DMSO concentration in the culture medium. No difference was observed between control and vehicle (EtOH and DMSO)-treated cells. Data shown are representative of three independent experiments. Significance of the differences between control (untreated cells) and RSV-, QCT-, API-, α-toco- or 7KC-treated cells; Mann–Whitney test: * *p* < 0.05 or less. Significance of the differences between 7KC-treated cells and (7KC + (RSV, QCT, API or α-toco))-treated cells; Mann–Whitney test: # *p* < 0.05 or less. No significant differences were found between control and vehicle-treated cells (Ethanol (EtOH): 0.2% and DMSO: 0.2%).

It is now well established in many cells that 7KC is a potent inducer of apoptosis and autophagy [29,30]. We described in human promonocytic U937 cells, murine microglial BV-2 cells and murine oligodendrocyte 158N cells, that 7KC induces a mode of cell death associated with oxidative stress and some characteristics of apoptosis and autophagy, named oxiapoptophagy [29,30]. In murine neuroblastoma N2a cells, it was therefore important to characterize the mode of cell death induced under treatment with 7KC in the presence or absence of RSV, QCT, API (3.125 µM), or of α-tocopherol (400 µM), used as reference for cytoprotection. In the present study, under treatment with 7KC (50 µM, 48 h), several cells with fragmented and/or condensed nuclei, characteristic of apoptotic cells were identified by fluorescence microscopy after nuclei staining with Hoechst 33342 (Figure 11A). This induction of apoptosis was biochemically characterized by a cleavage of caspase-3 (presence of the 17 kDa cleaved sub-unit), leading to the activation of caspase-3 supported by the fragmentation of PARP (presence of the 89 kDa cleaved fragment) (Figure 11B). Under treatment with 7KC, autophagy was characterized by the presence of several monodansyl cadaverine (MDC) positive vacuoles, considered as autophagic vacuoles detected by fluorescence microscopy [88] and by the conversion of LC3-I (18 kDa) to LC3-II (16 kDa) (increased ratio (LC3-II/LC3-I)) revealed by Western blotting (Figure 11B and Figure S7). By using rapamycin (an autophagy activator), cell death assessed by the FDA test was decreased (the number of positive FDA cells increased in the assay (7KC + rapamycin) compared to 7KC); on the other hand, in the presence of 3-methyladenine (3-MA, an autophagy inhibitor), cell death was increased (the number of positive FDA cells was decreased in the assay (7KC + 3-MA) compared to 7KC) suggesting that on N2a cells survival autophagy was activated by 7KC (Figure S8), When N2a cells were simultaneously treated with 7KC associated with RSV, QCT, API or α-tocopherol, apoptosis was inhibited and autophagy was normalized: the presence of cells with fragmented and/or condensed nuclei was strongly reduced as well as the presence of cells with several MDC positive vacuoles (Figure 11A and Figure S7). In addition, by Western blotting, when 7KC was added with RSV, QCT or API, the levels of cleaved caspase-3, fragmented PARP and LC3-II form were strongly reduced compared to 7KC alone (Figure 11B). In control and vehicle-treated cells (EtOH 0.2%; DMSO 0.2%) and in RSV-, QCT-, API- and α-tocopherol-treated cells no signs of apoptosis and autophagy were observed (Figure 11). Thus, in N2a cells, 7KC induces a mode of cell death simultaneously associated with apoptotic and autophagic criteria which is prevented by RSV, QCT, API or α-tocopherol.

## 4. Discussion

In the present study, the cytotoxicity of 7KC was characterized in neuronal N2a cells and the ability of polyphenols, abundant in the Mediterranean diet (RSV, QCT and API), to counteract the harmful effects of this oxysterol was determined. Preventing the cytotoxicity of 7KC with polyphenols could be a means of treating certain neurodegenerative diseases associated with oxidative stress resulting from increased levels of 7KC, such as AD [32] as well as other age-related diseases [28,31]. The results obtained with the three polyphenols (RSV, QCT and API) show that these molecules are oppose 7KC-induced oxiaoptophagy. Thus, RSV, QCT and API greatly reduce oxidative stress, organelle dysfunction (mitochondria, peroxisome), as well as the activation of apoptosis and autophagy.

As it was reported on murine 158N oligodendrocytes [40] and murine BV-2 microglial cells [45] that 7KC induced a mode of cell death by oxiapoptophagy [41,89], we characterized 7KC-induced cell death in murine neuronal N2a cells. In agreement with data obtained on nerve cells, 158N and BV-2 cells, but also on human promonocytic U937 cells [41] and bone marrow mesenchymal stem cells [46], our data demonstrate that 7KC induces an oxiapoptophagic mode of cell death in N2a cells. On these cells, cell death induced by 7KC was studied after 48 h of treatment in a range of concentrations from 1.5625 to 100 µM. While no toxicity was observed with 7KC (1.5625, 3.125, 6.25, 12.5 and 25 µM), significant cytotoxic effects were identified at 50 and 100 µM with two complementary tests: the FDA assay (evaluating esterase activity) and the SR101 assay (evaluating adherent cells) [16]. Under these conditions, the IC50 value of 7KC was 50 µM. So, the cell characteristics of 7KC-induced cell death were determined at this concentration.

In agreement with previous data obtained on other cell types, 7KC induced an important increase in plasma membrane permeability which could be the consequence of plasma membrane alteration due to oxidative stress (ROS overproduction measured with DHE) [40,45]. This important oxidative stress was associated with modifications of anti-oxidant enzymes (catalase, GPx1, SOD1 and SOD2): catalase activity was enhanced whereas SOD and GPx activities were decreased; GPx1 mRNAs level was decreased, whereas SOD1 and SOD2 mRNAs levels were enhanced; the protein levels of GPx1 and SOD1 were reduced, whereas those of SOD2 and catalase were enhanced. In addition, at the mRNA level, we also observed a down-regulation of the nuclear transcription factor Nrf2 which up-regulates numerous proteins contributing to prevention of damage due to oxidative stress [53,54].

Altogether, as 7KC is known to induce many organelle dysfunction consistent with its sub-cellular distribution [90]; this lead us to characterize the impact of 7KC at the mitochondrial and peroxisomal levels since such alterations have been described on several 7KC-treated glial and microglial cells. It is noteworthy that 7KC-induced loss of mitochondrial membrane potential (ΔΨm) was associated with a mitochondrial ROS overproduction identified with MitoSOX. In addition, under treatment with 7KC, a decrease in AMP-activated protein kinase alpha (AMPKα), sirtuin 1 (SIRT1) and peroxisome proliferator-activated receptor gamma coactivator-1α (PGC-1α) mRNAs encoding for proteins involved in mitochondrial biogenesis was also observed. However, at the protein level, SIRT1 and PGC1-α were decreased whereas AMPKα was unchanged. Such differences suggest different kinetics of synthesis and/or degradation between these proteins. As the mitochondria and peroxisome are tighly connected organelles [91,92], the impact of 7KC on the peroxisomal mass, biogenesis and activity was also studied. Thus, lower levels of the ABCD3 transporter, considered a peroxisomal mass marker taking into account the abundance and the size of peroxisomes [93], was observed by flow cytometry in 7KC-treated cells, as well as a decreased expresssion of genes associated with peroxisomal biogenesis (*Pex13*, *Pex14*) and peroxisomal β-oxidation (*Abcd1*, *Acox1*, *Mfp2*, *Thiolase A*) [85,87,94]. In addition, the protein levels of the peroxisomal transporter ABCD1 and of the peroxisomal enzyme MFP2 were reduced. These peroxisomal dysfunctions observed in N2a cells evocate those described in 158N murine oligodendrocytes and BV-2 microglial cells [40,45], suggesting that the deleterious effects of 7KC observed on these organelles do not depend on the type of nerve cells concerned (glial cells, microglial cells or neurons).

7KC-induced cell death also presented characteristic of apoptosis (cells with fragmented and/or condensed nuclei; cleaved caspase-3 and PARP fragmentation) associated with autophagic criteria (cells with monodansyl cadaverine positive vacuoles; activation of LC3-I to LC3-II). In 7KC-treated N2a cells, the conversion of LC3-I to LC3-II and/or the accumulation of LC3-labeled autophagosomes revealed by staining with monodansylcadaverine might be due to the blockade of this pathway at a later stage, as happens for some autophagy blockers such as chloroquine [95,96,97]. Furthermore, an increase in ROS generation has also been reported to ultimately prevent the fusion of lysosomes with autophagosomes and could therefore contribute to increase LC3-II level [95]. Altogether, our present results demonstrate that 7KC also induces a type of death by oxiapoptophagy on N2a cells and confirm in nerve cells that the type of autophagy activated by 7KC is survival autophagy, such as that described on 7KC-treated cells of the vascular wall (macrophages, smooth muscle cells) [43,98,99], as well as on 7β-hydroxycholesterol-treated rat C6 glioma cells [88]. Our data bring new evidence that 7KC-induced oxiapoptophagy is independent of the cell type and of the species considered, since it can be observed on monocytic cells [43], human bone marrow mesenchymal stem cells [46], murine glial and microglial cells [40,45] and also murine neuronal N2a cells. The fact that oxiapoptophagy is a highly conserved cell death from one type of cell to another has important consequences for preventing the age-related diseases associated with 7KC. Indeed, for the latter, molecules capable of opposing oxiapoptophagy could have a systemic effect and therefore a significant general impact on aging. In addition, as 7KC induces death by oxiapoptophagy in the three main cell types of the central nervous system (neuronal, microglial and glial cells) [29,30], identifying molecules which inhibit oxiapoptophagy should theorically allow neurodegeneration to be counteracted.

In our laboratory, in order to inhibit or reduce oxiapoptophagy induced by 7KC, the signaling pathways of which have been described and summarized by Vejux et al. [29] and Brahmi et al. [30], we have chosen a strategy based on the use of synthetic molecules, natural molecules or mixtures of molecules (oils, phenolic extracts) [30,100,101]. At the moment, few molecules are able to attenuate the cytotoxic effects of 7KC in vitro when treating cells either before the addition of 7KC, or simultaneously. Among the synthetic molecules are two activators of the Nrf2 pathway, dimethyl fumarate and its major metabolite, monomethyl fumarate, which have shown cytoprotective effects in 158N cells not only on 7KC but also on 7β-hydroxycholesterol, which can also be formed by auto- oxidation of cholesterol as well as enzymatically from 7KC via the enzyme 11β-HSD1 [55,56]. Some lipids, such as α-tocopherol (the main component of Vitamin E constituted of four tocopherols and four tocotrienols), oleic acid (C18:1 n-9: the main fatty acid of olive oil) and docosahexaenoic acid (C22:6 n-3: abundant in oily and blue fishes as salmon, trout, sardines, herrings, mackerel and tuna) also have important cytoprotective activities against 7KC in different cell types, including nerve cells; they strongly reduce oxidative stress, mitochondrial dysfunction (loss of ΔΨm), peroxisomal changes, apoptosis and autophagy [29,30,93,102]. It has been clearly established that α-tocopherol prevents the accumulation of 7KC in the lipid rafts, inhibiting thus the cascade of events leading to cell death [68,103,104]. It was also reported that argan, olive and milk thistle seed oils are capable of counteracting 7KC-induced oxiapoptophagy [105,106]. However, there is no study on the effects of polyphenols on oxiapoptophagy. It has been reported on ARPE-19 retinal epithelial cells that RSV attenuates 7KC-induced cell death [21]. RSV also counteracts inflammation in human M1 and M2 macrophages upon challenge with 7KC [107], and in C2C12 mouse skeletal muscle cells, white mustard and coriander extracts rich in polyphenols have shown antioxidant activities [108]. In addition, in intestinal cells, an oxysterol mixture composed of 42.96% 7KC, 32.3%, 5α,6α-epoxycholesterol, 5.76% 5β,6β-epoxycholesterol, 4.26% 7α-hydroxycholesterol, and 14.71% 7β-hydroxycholesterol, to a final concentration of 60 μM induces inflammation and oxidative stress which are prevented by olive oil phenolic extracts which directly modulate p38 and c-Jun N-terminal kinase (JNK)1/2 phosphorylation and activation of NF-kB; the phenolic extract also inhibited inducible Nitrous Oxide Synthase (iNOS) induction, keeping NO concentration at the control level [58,59]. The present study demonstrates for the first time cytoprotective activities of some polyphenols (RSV, QCT and API) present in the Mediterranean diet on 7KC-induced oxiapoptophagy, and confirms the potential of polyphenols in prevention of 7KC-induced cytotoxicity. The cytoprotective effects of RSV, QCT and API were studied at 3.125 and 6.25 µM because at higher concentrations (from 12.5 µM) RSV and QCT, in agreement with previous results, activate the death of N2a cells [16]. These cytotoxic effects are probably related to the anti-tumor activities of these two polyphenols observed for concentrations close to 50 µM [109,110]. In contrast, API up to 100 µM does not induce cell death. Despite these differences in toxicity, at concentrations of 3.125 and 6.25 µM, RSV, QCT and API have similar cytoprotective effects against 7KC. Thus, RSV, QCT and API similarly attenuate oxidative stress (ROS overproduction at whole cell level and at mitochondrial level, down regulation (GPx1, Nrf2) and up-regulation (SOD1, SOD2) of gene expression) and apoptosis (fragmentation and/or condensation of the nuclei; caspase-3 cleavage and PARP fragmentation) and prevent autophagy (induction of monodansyl cadaverine positive vacuole formation; activation of LC3-I to LC3-II). Since 7KC-induced survival autophagy associated with mitophagy, pexophagy and reticulophagy, autophagy could be considered a consequence of oxidative stress, inhibiting oxidative stress by RSV, QCT and API would prevent the activation of autophagy and normalize its activity. The three polyphenols (RSV, QCT and API) are thus added to the short list of natural molecules (α-tocopherol, oleic acid (C18:1 n-9), docosahexaenoic acid (DHA, C22:6 n-3) and synthetic molecules (dimethyl fumarate, monomethyl fumarate) which efficiently prevent 7KC-induced oxiapoptophagy [29,30]. In addition, on N2a cells, RSV, QCT and API prevent 7KC-induced organelle dysfunction. In N2a cells, our data support that 7KC-induced apoptosis activates the mitochondrial pathway due to a marked loss of ΔΨm which is also prevented by RSV, QCT and API. As QCT, RSV and API also prevent the decreased gene expression and the decreased protein level of SIRT1 and PGC-1α implicated in the control of mitochondrial biogenesis, our data also suggest that 7KC-induced oxiapoptophagy reduces mitochondrial biogenesis and that the three polyphenols used counteract this effect. The ability of RSV, QCT and API to prevent 7KC-induced peroxisomal changes was also studied on N2a cells for the following reasons: i) it is now well established that mitochondrial dysfunction can impact peroxisomal activity, and thi occurs reciprocally [91,92], and ii) we previously reported in murine microglial BV-2 cells and in murine oligodendrocytes 158N cells that 7KC induced morphological, topographical and functional peroxisomal changes [41,44,45,76]. Our data clearly established that RSV, QCT and API counteract the decrease in ABCD3 (considered as a marker of peroxisomal mass) at the protein and mRNA levels, the decrease protein level of ABCD1 and MFP2 involved in the peroxisomal β-oxidation as well as the decreased expresssion of genes associated with peroxisomal biogenesis (*Pex13*, *Pex14*) and peroxisomal β-oxidation (*Abcd1*, *Acox1*, *Mfp2*, *Thiolase A*). In N2a cells, in agreement with data obtained on 158N murine oligodendrocytes and murine microglial BV-2 cells [44,76], our results underline that 7KC induces both mitochondrial and peroxisome dysfunction, which justifies paying more attention to these organelles, which could constitute new therapeutic targets in neurodegenerative diseases. As cytoprotective effects were observed with α-tocopherol, which also prevent 7KC-induced oxidative stress, apoptosis and autophagy on BV-2 and 158N cells [29,30], it is suggested that 7KC-induced oxiapoptophagy probably activates similar signaling pathways in 158N, BV-2 and N2a cells.

Hence, the three polyphenols studied (RSV, QCT and API) at concentrations in the range of 3 to 6 µM have significant cytoprotective activities towards 7KC. Nevertheless, when these polyphenols are provided by food or in the form of food supplements, they may be subject to more or less pronounced degradation by the gut microbiota [111]. Moreover, these polyphenols have to pass numerous barriers such as intestinal and hemato-encephalic barriers if we are to consider their tissue effects and in particular at the cerebral level. In addition, these molecules have a short plasma half life and are rapidly metabolized leading to the formation of glucurono, sulfo or tauro-conjugates [111]. While some of these secondary metabolites are inactive, others may have beneficial activities [112]; consequently, for each polyphenol, identifying stable and bioactive metabolites is of therapeutic interest. In vitro, alternative methods to animal experimentation (Lab on Chips), including several cell types in a dynamic context (microfluidic), would make it possible to specify whether polyphenols but especially their metabolites can efficiently pass a succession of different barriers and are active on defined target cells [29]. Studying the metabolism of polyphenols by nerve cells is also possible in vitro from primary cultures of glial, microglial and neuronal cells as well as using recognized nerve cell lines as models: 158N, BV-2, SK-N-BE, SH-SY5Y and N2a cells. These cell lines could also be used in co-cultures to evaluate, in the context of oxiapoptophagy, the impact of one cell type on another. At the moment, in order to preserve in vivo the in vitro activities of polyphenols, micro- and nano-encapsulation strategies that have already been proven (including at the level of the central nervous system) are possible [113,114,115,116,117]. In addition, as 7KC induces major organelle dysfunction (mitochondria, peroxisome), targeting of these organelles by functionalized nanoparticles could also be considered (Targeted Organelle Nano therapy: TORN therapy) to oppose the toxicity of 7KC [30,101]. Currently, there are several lines of evidence that new nanoformulations of polyphenols (RSV, QCT) strongly improve the bioavailability of these polyphenols in vivo [118,119] and this makes it possible to envisage therapeutic applications with these molecules. Using iron nanoparticles, this would have the advantage of implementing targeted therapies. One can also imagine synthesizing lipophilic cationic derivatives of RSV, QCT and API in order to target the mitochondria and to inhibit the dysfunctions (ROS overproduction, drop of ΔΨm) induced by 7KC. It is also possible that aza- or azo-stilbenes deriving from RSV, QCT and API may have greater therapeutic potential than the original natural molecules [120].

In conclusion, on murine N2a cells as well as on human SK-N-BE and SH-SY5Y cells, RSV, QCT and API used at low concentrations (3.125 and 6.25 µM) prevent 7KC (50 µM)-induced cell death. The cytoprotection with these polyphenols is more efficient in pre- and co-treatment than in post-treatment. In addition, on N2a cells, 7KC induces a mode of cell death by oxiapoptophagy which is attenuated by RSV, QCT and API reinforcing the interest in these polyphenols for the prevention of neurodegeneration. The oxidative stress as well as the mitochondrial and peroxisomal dysfunction associated with 7KC-induced oxiapoptophagy are strongly reduced. Since 7KC is associated with cardiovascular diseases, eye diseases (cataract, AMD) and bowel diseases [28,30,31,59], our data support that these polyphenols widely represented in the Mediterranean diet, which can be incorporated in food supplements, could be of interest to prevent and/or treat these age-related and chronic inflammatory diseases. However, from a pharmacological point of view, due to the limited bioavailability of polyphenols in vivo, chemical and galenic strategies should be considered and must be developed to preserve their efficacy. In addition, whereas RSV, QCT and API (used at 3.125 and 6.25 µM) are equivalent in terms of cytoprotection against 7KC, they have variable toxicities from concentrations of 12.5 µM and above. Indeed, while API is only slightly toxic at 50 and 100 µM, a marked induction of cell death was observed with RSV and QCT from 12.5 µM. This gives API an advantage in considering therapeutic applications. API also has a neurotrophic activity: antioxidant activities and induction of neuronal differentiation [16]. Given these characteristics, compared to RSV and QCT, which have cytoprotective activities in a reduced concentration range, API therefore presents a potentially more attractive profile for treatment of age-related diseases, including neurodegeneration.

## Supplementary Materials

The following are available online at https://www.mdpi.com/2073-4409/9/11/2346/s1, Figure S1: Measurement by spectrofluorescence of the concentration and time dependent uptake of polyphenols by N2a cells, Figure S2: Morphological evaluation of the protective effects of resveratrol, quercetin, apigenin, α-tocopherol and 7-ketocholesterol-induced cell death on N2a cells by phase contrast microscopy, Figure S3: Evaluation of the protective effects of resveratrol, quercetin, apigenin, α-tocopherol and 7-ketocholesterol-induced cell death on SK-N-BE cells determined with the fluoresceine diacetate assay, Figure S4: Evaluation of the protective effects of of resveratrol, quercetin, apigenin or α-tocopherol on 7-ketocholesterol-induced cell death on SH-SY5Y neuronal cells determined with the fluoresceine diacetate assay, Figure S5: Comparison of pre-treatment and post-treatment versus co-treatment with resveratrol, quercetin, apigenin and α-tocopherol on 7-ketocholesterol-induced cytotoxicity evaluated with the fluoresceine diacetate assay, Figure S6: Schematic representation of a peroxisome illustrating the peroxisomal β-oxidation, Figure S7: Visualization of autophagic vacuoles by staining with monodansyl cadaverine, Figure S8: Evaluation of the type of autophagy (lethal or survival) induced by 7-ketocholesterol on murine neuroblastoma N2a cells.
