# Supplementary material for: Prevention by Dietary Polyphenols (Resveratrol, Quercetin, Apigenin) Against 7-Ketocholesterol-Induced Oxiapoptophagy in Neuronal N2a Cells: Potential Interest for the Treatment of Neurodegenerative and Age-Related Diseases
Source: Cells. 2020 Oct 23;9(11):2346. doi: 10.3390/cells9112346 (PMC7690753; doi:10.3390/cells9112346)
Supplement: Supplementary file 1 [file cells-09-02346-s001.pdf]

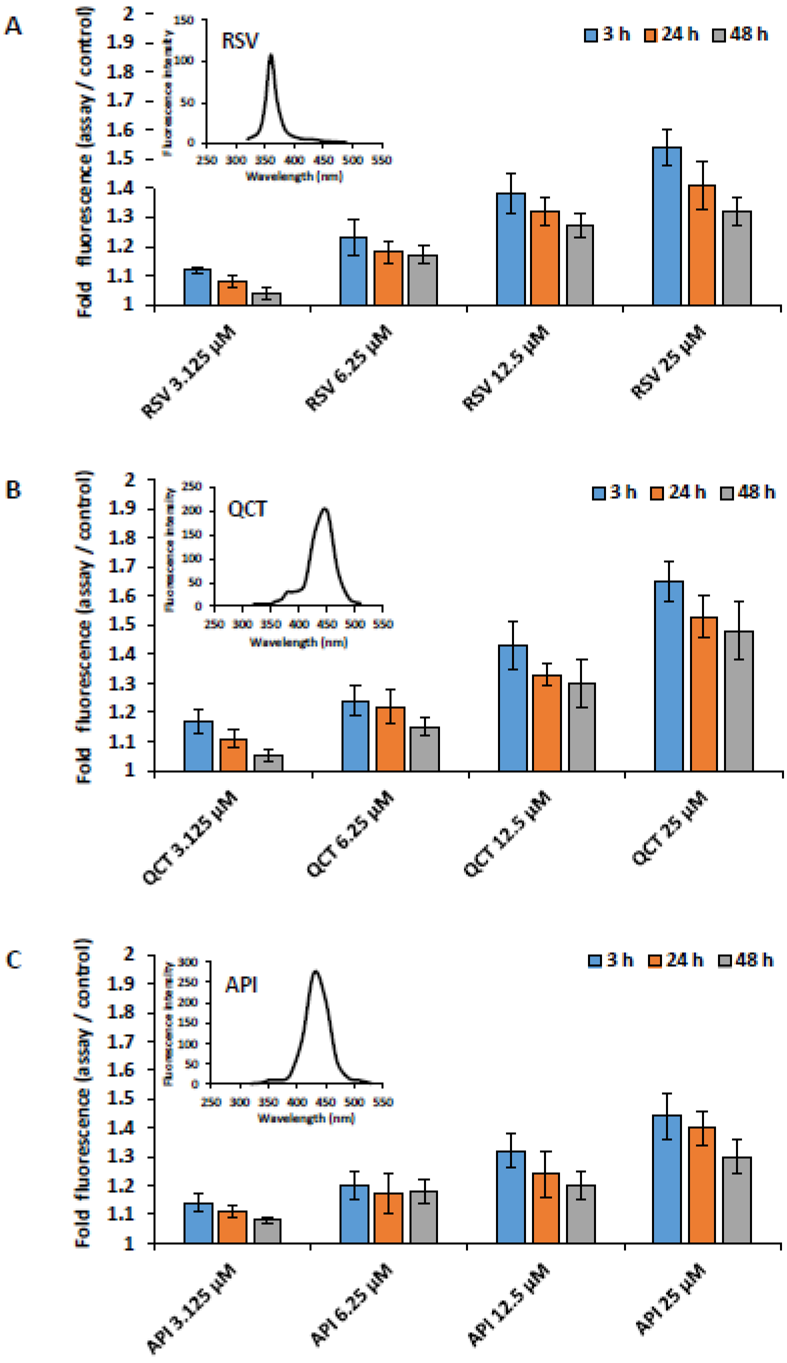

Supplementary Figure 1: Measurement by spectrofluorescence of the concentration and time dependent uptake of polyphenols by N2a cells. The uptake of RSV, QCT and API (based on the fluorescence properties of polyphenols) were determined by fluorescence measurements performed on N2a cells incubated with the polyphenols at various concentrations (3.125, 6.25, 12.5 and 25 µM) and times (3, 24 and 48 h). Each value is the mean ± standard deviation (SD) of three independent experiments. With the different polyphenols used (RSV, QCT and API), the fluorescent increases were observed after 3 h of treatment with the different concentrations used.

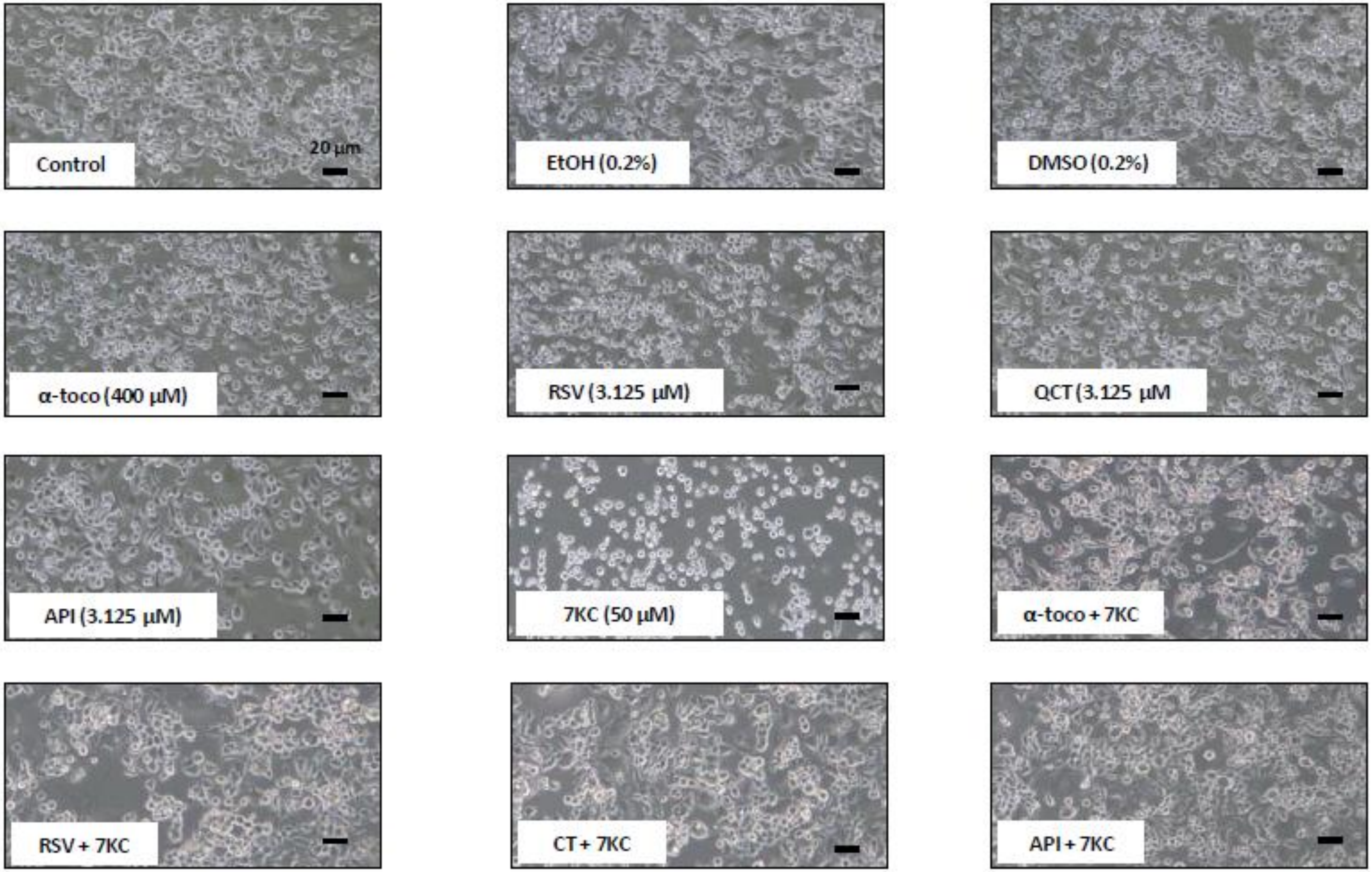

<u>Supplementary Figure 2:</u> Morphological evaluation of the protective effects of resveratrol, quercetin, apigenin, and α-tocopherol on 7-ketocholesterol-induced cell death on N2a cells by phase contrast microscopy. N2a cells were cultured for 48 h with or without 7-ketocholesterol (7KC, 50 µM) in the presence or absence of α-tocopherol (α-toco: 400 µM) used as positive control for cytoprotection, or with polyphenols: resveratrol (RSV), quercetin (QCT) or apigenin (API) used at 3.125 µM. Round cells considered as dead cells were observed in 7KC-treated cells. The loss of cell adhesion induced by 7KC was attenuated by RSV, QCT and API. Morphologically similar cells were observed in untreated cells (control), vehicles (EtOH, DMSO)-treated cells, polyphenols (RSV, QCT or API)- and α-topherol (α-toco)-treated cells. Observations were realized by phase contrast microscopy.

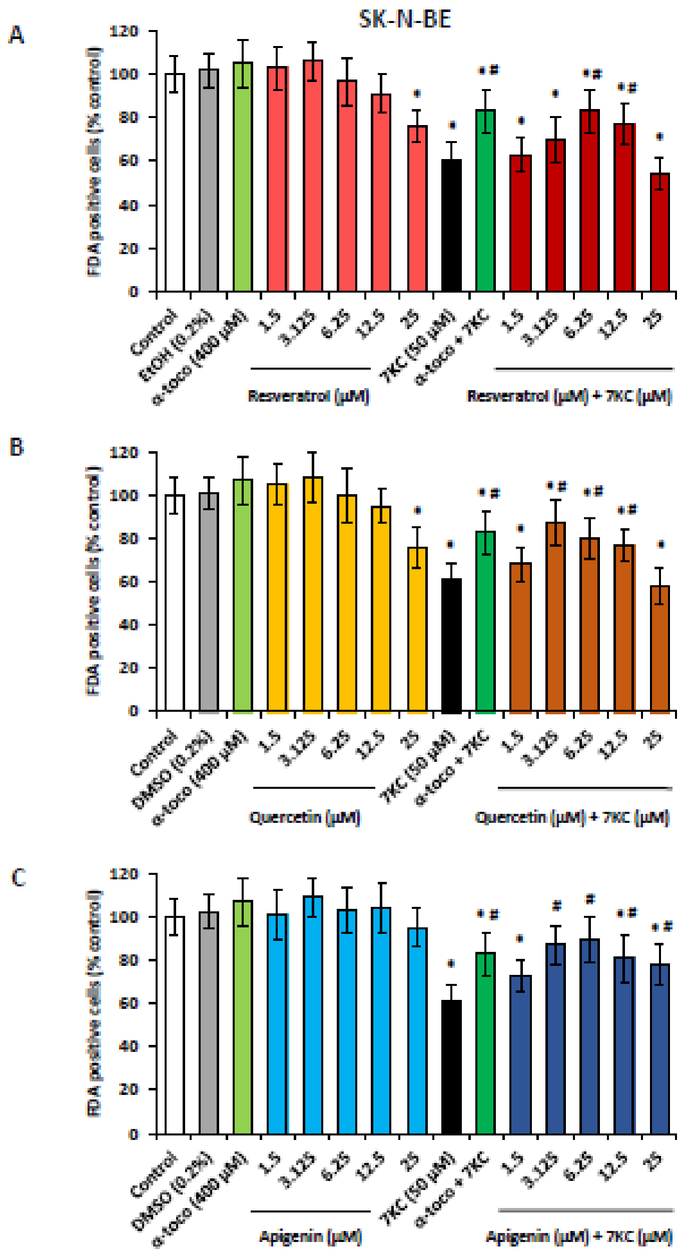

<u>Supplementary Figure 3:</u> **Evaluation of the protective effects of resveratrol, quercetin, apigenin, α-tocopherol and 7-ketocholesterol-induced cell death on SK-N-BE cells determined with the fluoresceine diacetate assay.** To evaluate the effects of polyphenols with or without 7-ketocholesterol (7KC) on cell viability of SK-NB-E cells, staining with fluorescein diacetate (FDA) was carried out on the cells treated for 48 h with or without 7KC (50 µM), in the presence or absence of resveratrol (RSV) **(A)**, quercetin (QCT) **(B)** and apigenin (API) **(C)** (1.5625 to 25 µM) or α-tocopherol (400 µM). Two vehicle controls were realized: EtOH (0.2%) used with RSV and 7KC, and DMSO (0.2%) used with QCT and API. Each value is the mean ± standard deviation (SD) of four independent experiments. Significance of the differences between control (untreated cells) and RSV-, QCT-, API-, α-toco- or 7KC-treated cells; Mann Whitney test: * P<0.05 or less. Significance of the differences between 7KC-treated cells and (7KC + (RSV, QCT, API or α-toco)) - treated cells; Mann Whitney test: # P<0.05 or less. No significant differences were found between control and vehicle-treated cells (Ethanol (EtOH): 0.2% and DMSO: 0.2%).

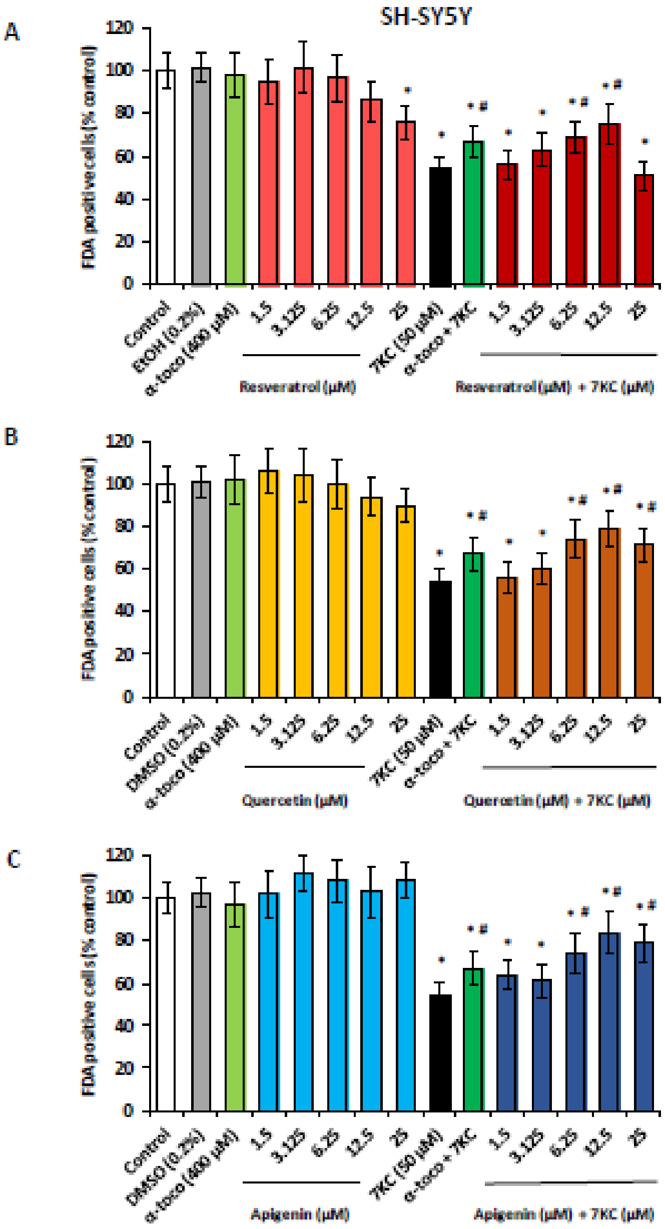

__Supplementary Figure 4:__ **Evaluation of the protective effects of of resveratrol, quercetin, apigenin or α-tocopherol on 7-ketocholesterol-induced cell death on SH-SY5Y neuronal cells determined with the fluoresceine diacetate assay.** To evaluate the effects of polyphenols with or without 7-ketocholesterol (7KC) on cell viability of SH-SY5Y cells, staining with fluorescein diacetate (FDA) was carried out on the cells treated for 48 h with or without 7KC (50 µM), in the presence or absence of resveratrol (RSV) **(A)**, quercetin (QCT) **(B)** and apigenin (API) **(C)** (1.5625 to 25 µM) or α-tocopherol (400 µM).Two vehicle controls were realized: EtOH (0.2%) used with RSV and 7KC, and DMSO (0.2%) used with QCT and API. Each value is the mean ± standard deviation (SD) of four independent experiments. Significance of the differences between control (untreated cells) and RSV-, QCT-, API-, α-toco- or 7KC-treated cells; Mann Whitney test: * P<0.05 or less. Significance of the differences between 7KC-treated cells and (7KC + (RSV, QCT, API or α-toco)) - treated cells; Mann Whitney test: # P<0.05 or less. No significant differences were found between control and vehicle-treated cells (Ethanol (EtOH): 0.2% and DMSO: 0.2%).

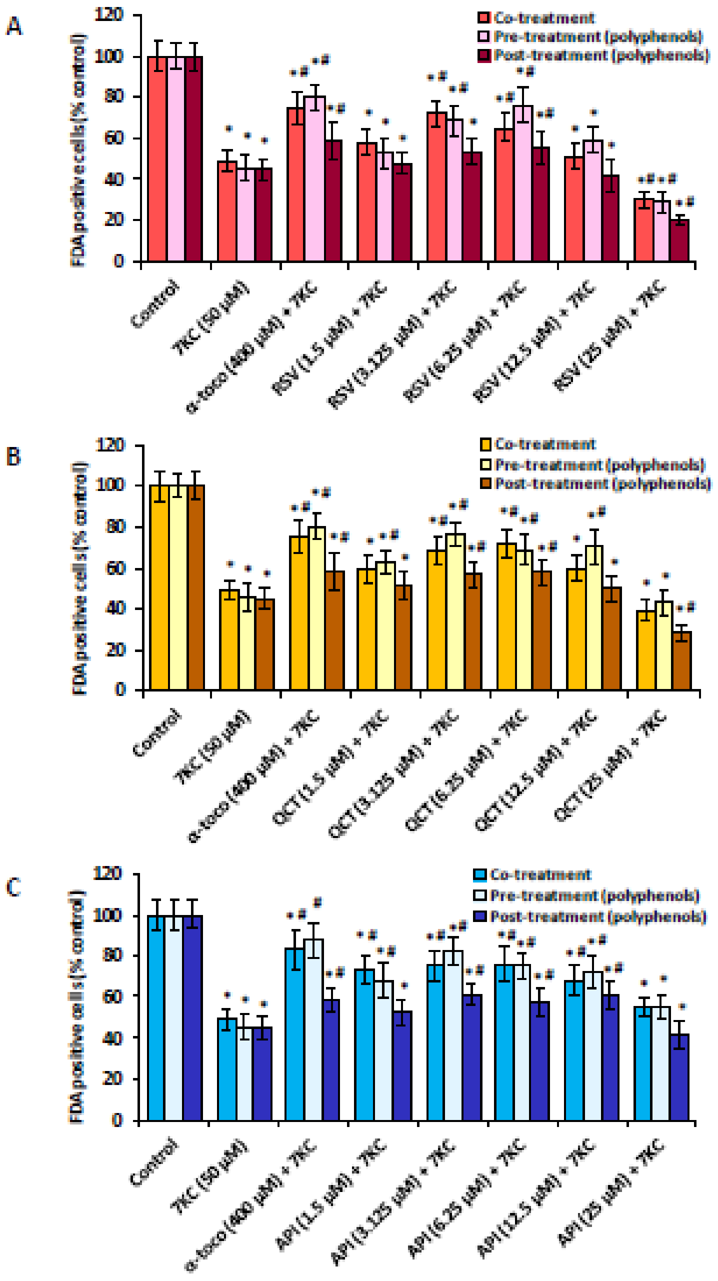

Supplementary Figure 5: Comparison of pre-treatment and post-treatment versus co-treatment with resveratrol, quercetin, apigenin and α-tocopherol on 7-ketocholesterol-induced cytotoxicity evaluated with the fluoresceine diacetate assay. N2a cells previously cultured for 24h were further pre-treated for 2h with resveratrol (RSV) **(A)**, quercetin (QCT) **(B)** and apigenin (API) **(C)** used at concentrations ranging from 1.5625 to 25 µM or with α-tocopherol (400 µM) (used as positive control) prior to 7-ketocholesterol (7KC, 50 µM) and were then incubated for an additional 48 h period of time. N2a cells were also post-treated with RSV, QCT and API; in those conditions, N2a cells previously cultured for 24h were further treated with 7KC (50 µM) and after 2 h, RSV, QCT and API were added for an additional 48 h period of time. Data obtained with cells simultaneously treated with 7KC and polyphenols are also shown. Cell viability was measured with fluoresceine diacetate (FDA) assay and presented as percentage of control. Each value is the mean ± standard deviation (SD) of three independent experiments. Significance of the differences between control (untreated cells) and RSV-, QCT-, API-, α-toco- or 7KC-treated cells; Mann Whitney test: * P<0.05 or less. Significance of the differences between 7KC-treated cells and (7KC + (RSV, QCT, API or α-toco)) - treated cells; Mann Whitney test: # P<0.05 or less.

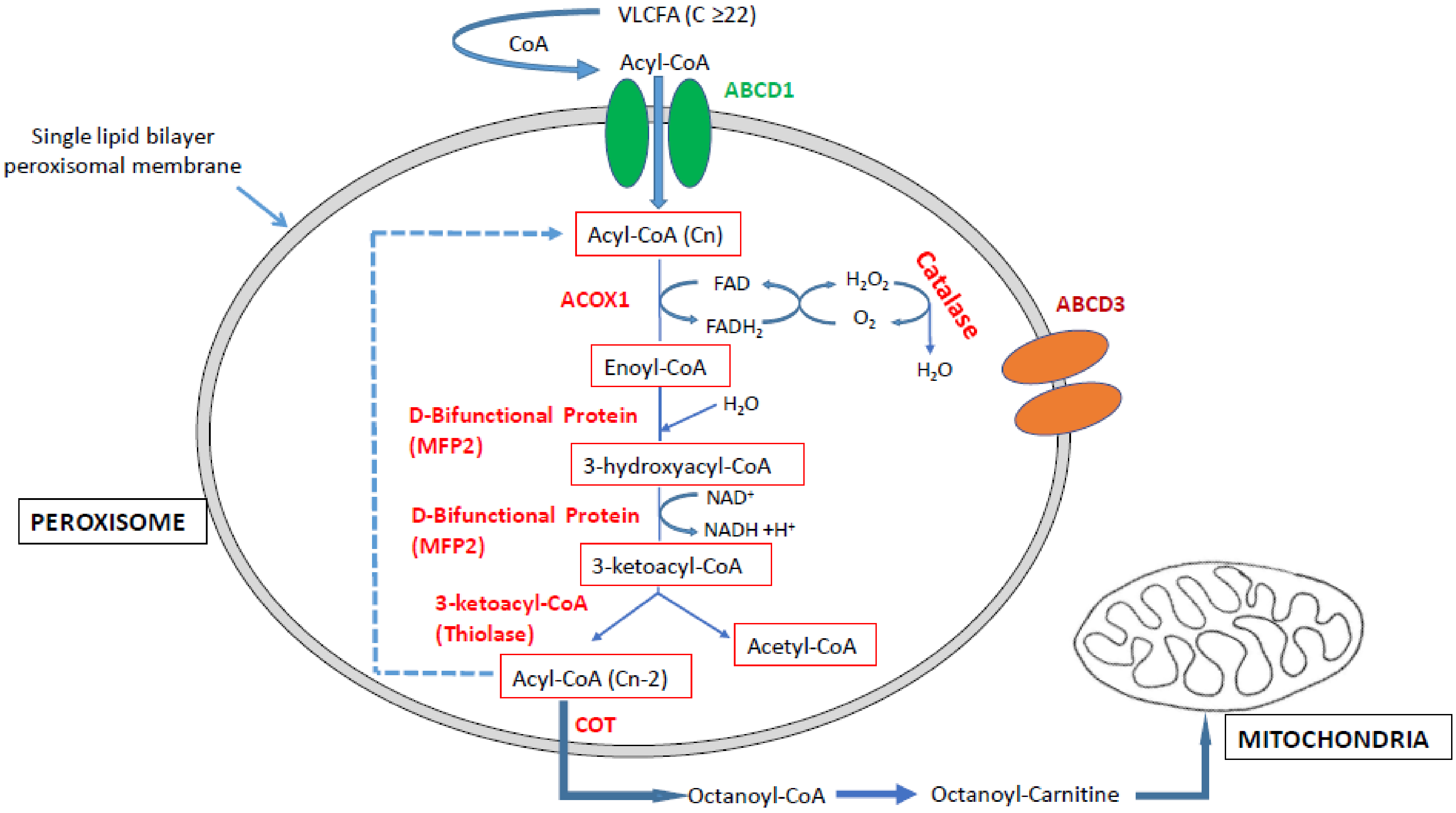

Supplementary Figure 6: Schematic representation of a peroxisome illustrating the peroxisomal β-oxydation. Very long chain fatty acid (VLCFA; C≥22) are activated by CoA to give Acyl-CoA, which is imported into the peroxisome by ABCD1. The peroxisomal β-oxidation which successively involves the enzymes ACOX1, MFP2 and thiolase allows to reduce the length of VLCFA from 2 carbons at each cycle of β-oxidation and gives (Acyl-CoA n-2) and Acetyl CoA; the final product of peroxisomal β-oxidation is octanoyl-CoA which is transported from the peroxisome to the cytosol via carnitine O-octanoyltransferase (CrOT enzyme) for further oxidation in the mitochondria in acetyl CoA **[87]**. Two different peroxisomal 3-ketoacyl-CoA thiolases for very-long-straight-chain fatty acids (thiolase A, ThA, and thiolase B, ThB) have been cloned in rodents, while only one corresponding gene (peroxisomal 3-acetyl-CoA acetyltransferase-1) has been identified in humans. Thiolase A, which is the active form of the enzyme in peroxisomal β-oxidation in rodent, has therefore been studied in N2a cells. ABCD3, which is a peroxisomal mass marker, present at the level of the peroxisomal membrane is also represented **[102]**. For reasons related to the scheme, the peroxisome appears larger than the mitochondria, whereas in reality the size of the peroxisome is much smaller than that of the mitochondria and in the range of 0.1 to 1 µm **[86]**. COT: carnitine octanoyl transferase.

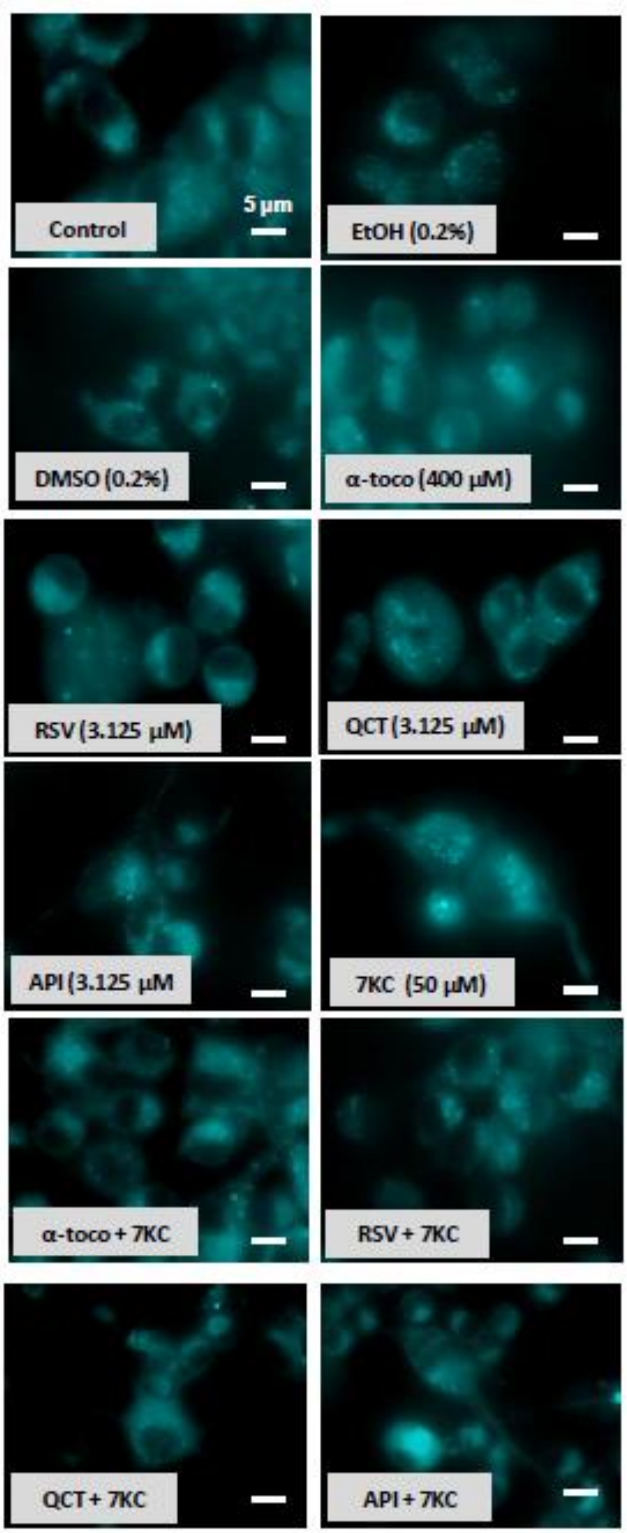

Supplementary Figure 7: Visualization of autophagic vacuoles by staining with monodansyl cadaverine. N2a cells were cultured for 48 h with or without 7-ketocholesterol (7KC, 50 µM) in the presence or absence of α-tocopherol (α-toco: 400 µM) used as positive control for cytoprotection, or with polyphenols: resveratrol (RSV), quercetin (QCT) or apigenin (API) used at 3.125 µM. Cells with several MDC positive vesicles considered as autophagic vacuoles were identified under treatment with 7KC; when 7KC was associated with RSV, QCT and API, the presence of cells with MDC vesicles was strongly reduced. Few cells with several MDC vesicles were present in control (untreated cells), vehicle-treated cells (EtOH 0.2%; DMSO 0.2%) and RSV-, QCT- and API-treated cells. No differences were observed between control and vehicle-treated cells (Ethanol (EtOH): 0.2% and DMSO: 0.2%).

A

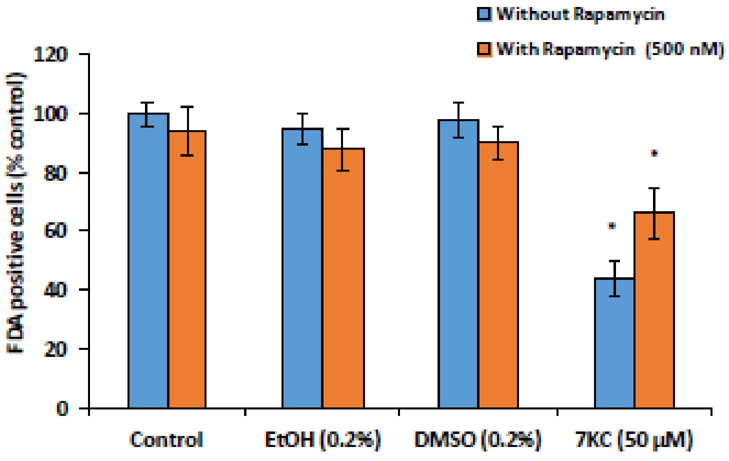

B

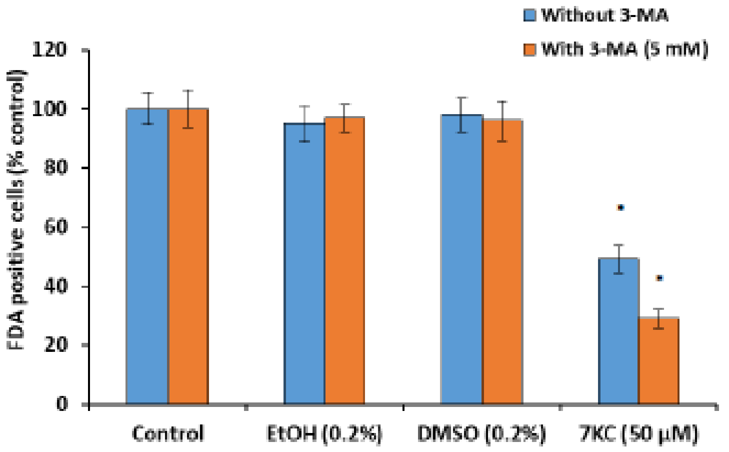

Suplementary Figure 8: Evaluation of the type of autophagy (lethal or survival) induced by 7-ketocholesterol on murine neuroblastoma N2a cells. N2a cells previously cultured for 24h, were further cultured with or without 7-ketocholesterol (7KC, 50 µM) in the presence or absence of rapamycin (inducer of autophagy) used at 500 nM (**A**) or 3-methyladenine (3-MA, autophagy inhibitor) used at 5 mM (**B**) for 48 h. The effects on cell viability were evaluated with the FDA assay. Data shown are representative from three independent experiments. Significance of the differences between control (untreated cells) and rapamycin, 3-MA or 7KC-treated cells; Mann Whitney test: * P<0.05 or less. Significance of the differences between 7KC-treated cells and (7KC + (rapamycin or 3-MA)) - treated cells; Mann Whitney test: # P<0.05 or less. No significant differences were found between control and vehicle-treated cells (Ethanol (EtOH): 0.2% and DMSO: 0.2%).
